# Selection of epigenetically privileged HIV-1 proviruses during treatment with panobinostat and interferon-α2a

**DOI:** 10.1016/j.cell.2024.01.037

**Published:** 2024-02-29

**Authors:** Marie Armani-Tourret, Ce Gao, Ciputra Adijaya Hartana, WeiWei Sun, Leah Carrere, Liliana Vela, Alexander Hochroth, Maxime Bellefroid, Amy Sbrolla, Katrina Shea, Theresa Flynn, Isabelle Roseto, Yelizaveta Rassadkina, Carole Lee, Francoise Giguel, Rajeev Malhotra, Frederic D. Bushman, Rajesh T. Gandhi, Xu G. Yu, Daniel R. Kuritzkes, Mathias Lichterfeld

**Affiliations:** 1 Ragon Institute of MGH, MIT and Harvard, Cambridge, MA 02139, USA; 2 Division of Infectious Diseases, Brigham and Women’s Hospital, Harvard Medical School, Boston, MA 02115, USA; 3 Division of Infectious Diseases, Massachusetts General Hospital, Boston, MA 02114, USA; 4 Department of Microbiology, University of Pennsylvania, Philadelphia, PA 19104, USA; 5 Division of Cardiology, Massachusetts General Hospital, Boston, MA 02114, USA

**Keywords:** HIV-1, integration sites, shock and kill, epigenetics, viral reservoir, interferon, innate immunity, HIV-1 cure, histone acetylation, panobinostat

## Abstract

CD4^+^ T cells with latent HIV-1 infection persist despite treatment with antiretroviral agents and represent the main barrier to a cure of HIV-1 infection. Pharmacological disruption of viral latency may expose HIV-1-infected cells to host immune activity, but the clinical efficacy of latency-reversing agents for reducing HIV-1 persistence remains to be proven. Here, we show in a randomized-controlled human clinical trial that the histone deacetylase inhibitor panobinostat, when administered in combination with pegylated interferon-α2a, induces a structural transformation of the HIV-1 reservoir cell pool, characterized by a disproportionate overrepresentation of HIV-1 proviruses integrated in ZNF genes and in chromatin regions with reduced H3K27ac marks, the molecular target sites for panobinostat. By contrast, proviruses near H3K27ac marks were actively selected against, likely due to increased susceptibility to panobinostat. These data suggest that latency-reversing treatment can increase the immunological vulnerability of HIV-1 reservoir cells and accelerate the selection of epigenetically privileged HIV-1 proviruses.

## Highlights

• Panobinostat and interferon-α2a stimulated viral transcription and innate immune cells
• The frequency of non-clonal intact proviruses was significantly reduced
• Proviruses in ZNF genes and in centromeric satellite DNA persisted during treatment
• Proviruses in proximity to H3K27ac histone modifications were selected against

## Graphical abstract

**Figure:**
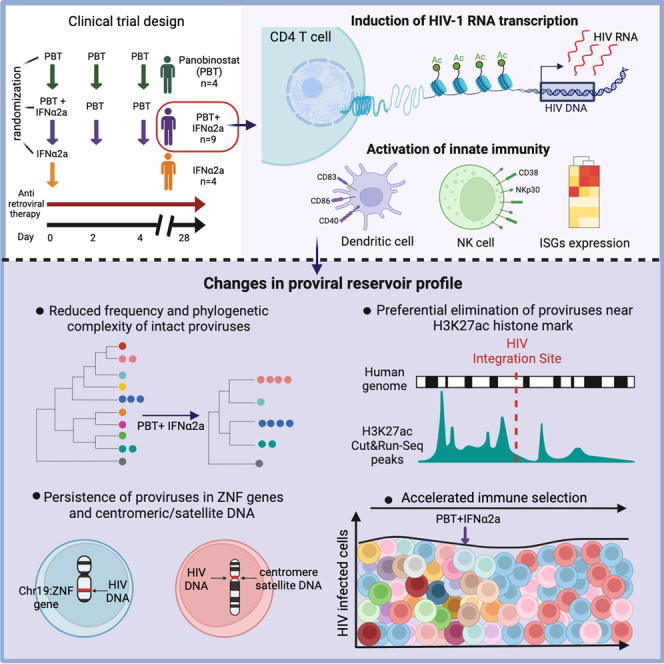

## Teaser

In a human clinical trial, combined treatment with the histone deacetylase inhibitor panobinostat and pegylated interferon-α2a increased the immunological vulnerability of HIV-1 reservoir cells and amplified naturally occurring immune selection pressure against HIV-1 proviruses integrated in permissive chromatin locations.

## Introduction

Life-long persistence of HIV-1 infection is primarily due to small numbers of virally infected, long-lasting memory CD4^+^ T cells that harbor chromosomally integrated HIV-1 DNA as a “provirus.”^1^^,^^2^ Within these cells, HIV-1 frequently remains in a transcriptionally silent (latent) state, which shields the virus from host immune responses and reduces viral cytopathic effects.^3^ Currently available antiretroviral agents effectively prevent new productive cycles of viral infection but have no activity against latently infected cells, which persist indefinitely and, for that reason, are typically referred to as “viral reservoir cells.” Most virally infected cells encountered in persons receiving antiretroviral therapy (ART) harbor genetically defective proviruses bearing lethal mutations and/or deletions,^4^^,^^5^^,^^6^^,^^7^ rendering them unable to drive viral rebound in case of treatment interruptions; by contrast, genome-intact, rebound-competent proviruses are only detected in a small minority of all virally infected cells.

Targeting HIV-1 reservoir cells in persons living with HIV-1 by pharmacological or immunological approaches has turned out to be extremely difficult, and except for toxic allogeneic hematopoietic stem cell transplantations,^8^ no interventions have been identified so far that effectively reduce the number of these cells in people living with HIV-1.^9^ Nevertheless, there is evidence that the human immune system is able to engage viral reservoir cells and that it can, at least in a subset of persons, exert considerable immune selection pressure on them. Such selection effects are arguably most apparent in elite controllers, who maintain undetectable levels of HIV-1 replication in the absence of treatment; in these persons, intact HIV-1 proviruses seem strongly enriched for integration in highly specific chromosomal locations in heterochromatin, presumably as a result of host immune responses that have cleared HIV-1 integrated in more accessible and permissive euchromatin.^10^^,^^11^ However, footprints of such an immune selection process can also be observed in the chromosomal integration site profile of ART-treated individuals,^12^^,^^13^ particularly after prolonged durations of antiretroviral therapy.^14^^,^^15^ The immune mechanisms driving this selection process are currently unknown, but a number of prior studies suggest a link between innate immune responses and viral reservoir dynamics.^16^^,^^17^^,^^18^^,^^19^^,^^20^

One approach to enhance the immunological vulnerability of HIV-1 reservoir cells focuses on the administration of pharmacological latency-reversing agents, which are designed to increase proviral transcriptional activity and might expose virally infected cells to host immune recognition.^21^ Such interventions, colloquially referred to as “shock and kill” approaches, have successfully led to proviral transcriptional reactivation in prior human clinical trials, specifically when histone deacetylase inhibitors (HDACis) were tested.^22^^,^^23^ These agents increase histone acetylation in selected chromatin regions of the human genome, which can translate into increased proviral transcriptional activity and, in specific cases,^24^ has resulted in small blips of plasma viremia during otherwise suppressive antiretroviral therapy; however, prior studies have failed to demonstrate a notable quantitative or qualitative change in the HIV-1 reservoir cell pool as a result of such interventions. Combinations of HDACi with immunological interventions, including therapeutic vaccines^25^^,^^26^ or broadly neutralizing antibodies,^27^^,^^28^^,^^29^ have been tested in an effort to enhance immune clearance of cells following viral latency disruption but did not lead to detectable changes in the proviral reservoir profile, as determined by standard assays for viral reservoir cell quantification.

In this manuscript, we describe a randomized-controlled human clinical trial in which panobinostat, a potent HDACi,^23^^,^^30^ was evaluated in combination with pegylated interferon (IFN)-α2a (PEG-IFN-α2a). PEG-IFN-α2a, mostly recognized for its prior clinical use for treatment of chronic hepatitis C and B, acts as an innate immune modulator and was selected for this study based on earlier investigations indicating that the pharmacological manipulation of type I IFN responses can reverse viral latency,^31^ alter viral reservoir dynamics,^32^ and influence HIV-1 rebound viremia during analytical treatment interruptions.^33^^,^^34^ We demonstrate that this combined study medication can increase proviral transcriptional activity, activate innate immune cells, and result in a trend for reducing the frequency of intact proviruses, as originally hypothesized. Interestingly, detailed evaluations of the proviral integration site landscape suggested selective elimination of proviruses integrated in chromatin regions loaded with acetylated H3K27ac histone modifications, which represent the primary molecular target sites for panobinostat. Together, these results provide proof-of-principle evidence that viral latency disruption with HDACi, in combination with innate immune activation, can transform the viral reservoir landscape through selective purging of proviruses integrated in proximity to acetylated histone marks.

## Results

### Study design, participants, and safety events

**Figure 1:**
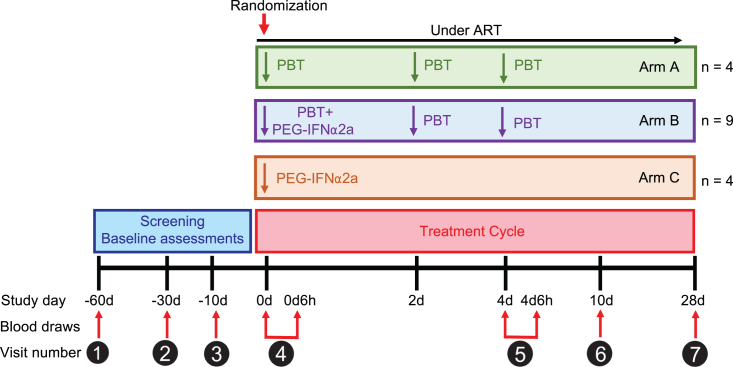
Schematic overview of the ACTIVATE study design Individual treatment arms after randomization are shown: arm A (n = 4) received only panobinostat (PBT), arm B (n = 9) received panobinostat (PBT) in combination with pegylated IFN-α2a (PEG-IFN-α2a), and arm C (n = 4) received only pegylated IFN-α2a. Time points of PBMC sample collection for analytical purposes are shown.

We conducted a prospective, open-label, randomized clinical trial with seventeen HIV-1-positive study participants receiving suppressive antiretroviral therapy, termed the ACTIVATE study (NCT02471430). Study participants were randomized to receive either panobinostat alone (arm A, n = 4 individuals), panobinostat in combination with PEG-IFN-α2a (arm B, n = 9 individuals), or PEG-IFN-α2a alone (arm C, n = 4 individuals) (Figure 1). Panobinostat was administered as an oral tablet at a dose of 15 mg on days 0, 2, and 4 for 1 week; PEG-IFN-α2a was administered as a single dose by subcutaneous injection of 180 μg on day 0. ART was continued throughout the entire treatment course in all study participants. Peripheral blood mononuclear cells (PBMCs) were collected for analytic purposes in all study participants on day 0 before treatment initiation, on day 4 (6 h after the last panobinostat dose), on day 28, and at other protocol-defined time points. Demographic and clinical characteristics of all study participants are summarized in Table S1. A total of 15 adverse events were observed in the study, most of which were grade 1 or 2. The most common adverse events observed for participants receiving PEG-IFN-α2a were body aches and fatigue. For participants receiving panobinostat, the most common adverse events were nausea and mild diarrhea. One grade 3 adverse event (self-limited neutropenia of 598 cells/μL) was observed in a participant receiving combined treatment; no serious adverse events were recorded (Table S2). The cohort reported here was preceded by study cohorts in whom the safety of the combined study medication was evaluated when panobinostat was administered at smaller doses (5 mg in step 1 and 10 mg in step 2) (Table S3). These two dose escalation steps, mandated by regulatory authorities and involving 8 participants in each step (6 receiving the combined treatment and 2 receiving panobinostat alone), did not reveal unexpected safety signals and supported an acceptable tolerability profile of the study medications (Tables S4 and S5).

### Increases in histone acetylation and viral transcription

**Figure 2:**
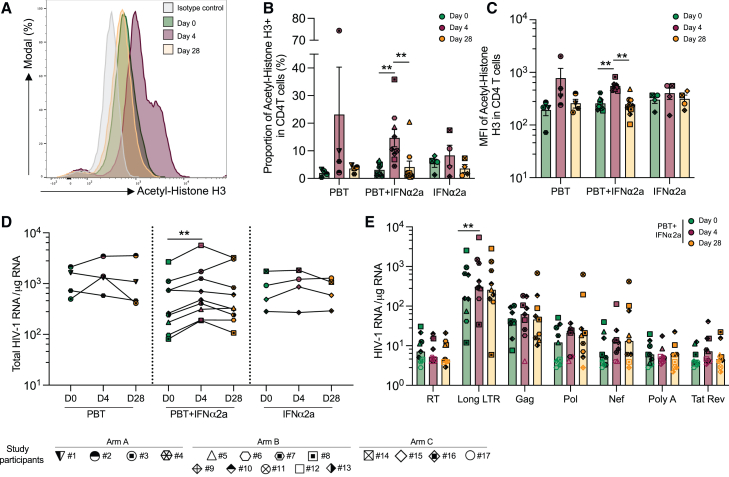
Increases in H3 histone acetylation and viral transcription during treatment with the study medication (A) Representative flow cytometry histograms of acetyl-histone H3 expression at days 0, 4, and 28 after panobinostat (PBT) treatment. (B) Proportion of CD4^+^ T cells expressing acetylated H3 in indicated study groups. (C) Mean fluorescent intensity (MFI) of acetylated H3 expression in CD4^+^ T cells in the different study groups. (D) Total cell-associated HIV-1 RNA copies (determined as the sum of the seven amplified HIV-1 transcripts) in purified CD4^+^ T cells normalized to 1 μg of cellular RNA and plotted on a log scale at day 0, day 4, and day 28. (E) Cell-associated HIV-1 RNA copies in purified CD4^+^ T from study participants receiving panobinostat and pegylated IFN-α2a cells normalized to 1 μg of cellular RNA. Seven different viral transcripts were analyzed: readthrough (RT), long LTR, Gag, Pol, Nef, polyA, and Tat/Rev. Empty symbols represent the limit of detection (LOD), defined as the total number of cells assayed without target identification. In (B) and (C), vertical bars reflect the mean with SEM; in (E), vertical bars reflect the geometric mean. ^∗^p < 0.05, ^∗∗^p < 0.01, Friedman test followed by Dunn’s multiple comparisons or Wilcoxon matched-pairs signed rank test. See also Figure S1.

**Figure S1:**
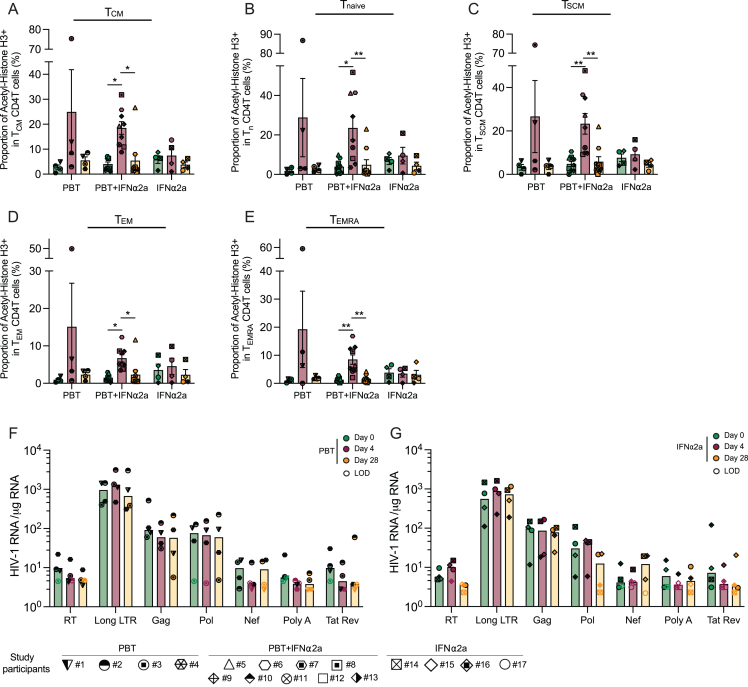
Changes in H3 acetylation and HIV-1 transcription in the ACTIVATE study, related to Figure 2 (A–E) Proportions of T_CM_ (A), T_naive_ (B), T_SCM_ (C), T_EM_ (D), and T_EMRA_ (E) expressing acetylated H3, determined by flow cytometry. Vertical bars reflect the mean with SEM. (F and G) HIV-1 RNA quantities in purified CD4^+^ T cells from study participants receiving panobinostat alone (study arm A in F) or pegylated IFN-α2a alone (study arm C in G) normalized to 1 μg of cellular RNA. Seven different HIV-1 transcripts were analyzed: readthrough (RT), long LTR, Gag, Pol, Nef, polyA, and Tat/Rev. Empty symbol represents the limit of detection (LOD) defined as the number of cells assayed without target identification. Vertical bars reflect the geometric mean. (^∗^p < 0.05, ^∗∗^p < 0.01, Friedman test followed by Dunn’s multiple comparisons or Wilcoxon matched-pairs signed rank test.)

We first studied the effect of panobinostat treatment on histone acetylation, using flow cytometry with antibodies recognizing acetylated histone 3 (H3) (Figure 2A). After 4 days of treatment with panobinostat alone or in combination with PEG-IFN-α2a, the proportions of acetylated histone H3-expressing CD4^+^ T cells increased in all study participants (mean increase of 4.7-fold from day 0 to 4, p = 0.0039); no significant increase in histone acetylation was observed during treatment with PEG-IFN-α2a alone (Figure 2B). An increase was also noted for the mean fluorescence intensity of antibodies recognizing acetylated H3 in recipients of the combined study medication (mean increase of 2.1-fold from day 0 to 4, p = 0.004) (Figure 2C). Increases in H3 acetylation following treatment with panobinostat occurred in all analyzed CD4^+^ T cell subpopulations at day 4 and returned to baseline at day 28 (Figures S1A–S1E). To evaluate the induction of HIV-1 transcription in response to panobinostat treatment, seven different types of CD4^+^ T cell-associated HIV-1 RNA transcripts were analyzed according to previously described protocols^35^^,^^36^; these transcripts define different steps in the HIV-1 gene expression cascade. We observed that total HIV-1 RNA (defined as the sum of all seven transcripts) increased for all the study participants receiving the combined treatment, except of one (mean increase of 1.83-fold, p = 0.0065) (Figure 2D). This increase was mostly attributable to elevated levels of long terminal repeat (LTR) transcripts, which increased by a mean of 1.85-fold (p = 0.0029) (Figure 2E), consistent with prior observations that panobinostat and other HDACis can support proviral transcriptional initiation.^37^^,^^38^ An increase in HIV-1 gag transcripts was noted in 5/9 study participants in the combined treatment group; this observation is in line with increased HIV-1 p24 protein levels detected in serum following panobinostat administration in some ART-treated participants of a prior human clinical trial.^39^ However, no significant changes were noted for more mature, multiply spliced, or poly-A HIV-1 transcripts, suggesting that the combination of panobinostat and PEG-IFN-α2a is not able to remove transcriptional blocks during transcriptional elongation and splicing. No significant differences in HIV-1 transcription were observed for participants who received panobinostat or PEG-IFN-α2a only (Figures S1F and S1G). In two study participants, both from arm A, plasma viremia blips of 25 and 213 HIV-1 RNA copies/mL were detected at 6 h after the first panobinostat dose; in one additional participant, again from group A, a plasma viral blip of 30 copies/mL was detected on day 4, 6 h after the final panobinostat dose.

### Activation of innate immune responses

**Figure 3:**
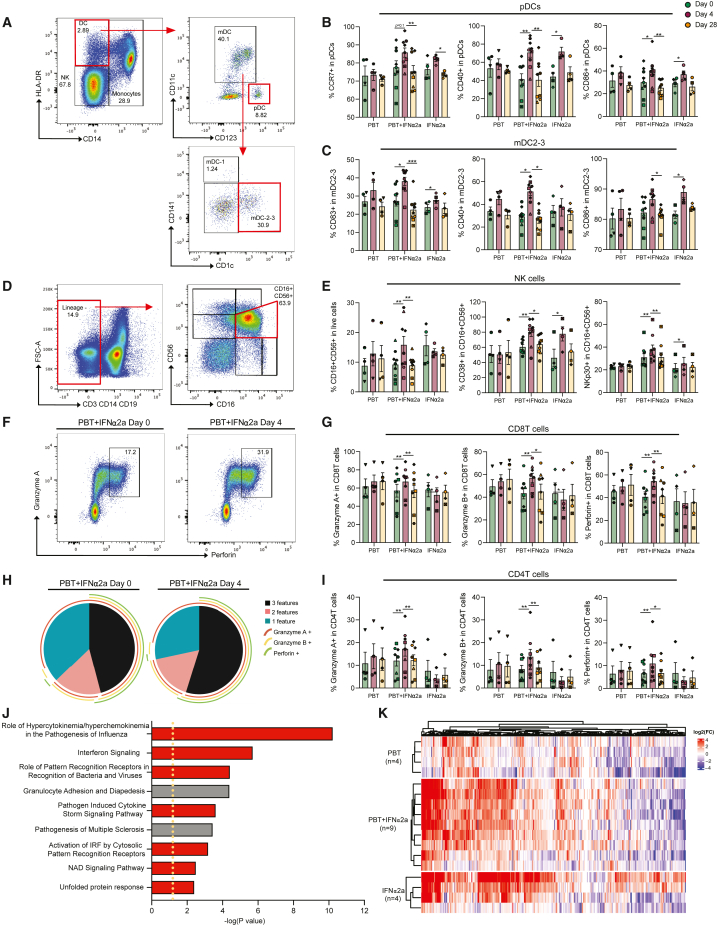
Innate immune responses during treatment with panobinostat (PBT) and/or pegylated IFN-α2a (A) Representative flow cytometry dot plots indicating the gating strategy for defining plasmacytoid dendritic cells (pDCs) and myeloid dendritic cells (mDCs). (B and C) Proportions of plasmacytoid dendritic cells (pDCs) (B) expressing CCR7 (left), CD40 (middle), and CD86 (right) or myeloid dendritic cells (mDCs) (C) expressing CD83 (left), CD40 (middle), and CD86 (right). (D) Representative flow cytometry dot plots indicating the gating strategy for defining natural killer (NK) cells and the subpopulations of cytotoxic NK cells expressing CD16 and CD56. (E) Proportions of cytotoxic NK cells (left) and the proportions of these cytotoxic NK cells expressing CD38 (middle) and NKp30 (right). (F) Representative flow cytometry dot plots indicating the gating strategy for defining CD8^+^ T cells co-expressing perforin and granzyme A at day 0 (left) and day 4 (right). (G) Proportions of CD8^+^ T cells expressing granzyme A (left), granzyme B (middle), and perforin (right). (H) Simplified presentation of incredibly complex evaluations (SPICE) diagrams reflecting the proportions CD8^+^ T cells expressing granzyme A, granzyme B, and/or perforin at day 0 before treatment or at day 4 after receiving the combined treatment. The pie charts indicate the relative proportions of cells expressing 1, 2, or 3 features; individual features are shown as overlaying arches. (I) Proportion of CD4^+^ T cells expressing granzyme A (left), granzyme B (middle), and perforin (right). (J) Significant canonical pathways predicted by ingenuity pathway analysis (IPA) of differentially expressed genes (DEGs) (day 4 vs. day 0) using RNA-seq data from CD4^+^ T cells of participants receiving the combined treatment. Pathways predicted to be upregulated are marked in red, and pathways with no predicted directional change are marked in gray. The cutoff was established at −log (p value) ≥ 1.3 (yellow dashed line). (K) Heatmap displaying interferon regulated gene (IRG, defined by www.interferome.org), differentially expressed at day 4 vs. day 0, determined using RNA-seq data from CD4^+^ T cells of individuals receiving the combined study medication. In (B), (C), (E), (G), and (I), vertical bars reflect the mean with SEM. ^∗^p < 0.05, ^∗∗^p < 0.01, Friedman test followed by Dunn’s multiple comparisons was used for all the comparisons. Symbols for identification of individual study participants are as in Figure 2. See also Figure S2.

**Figure S2:**
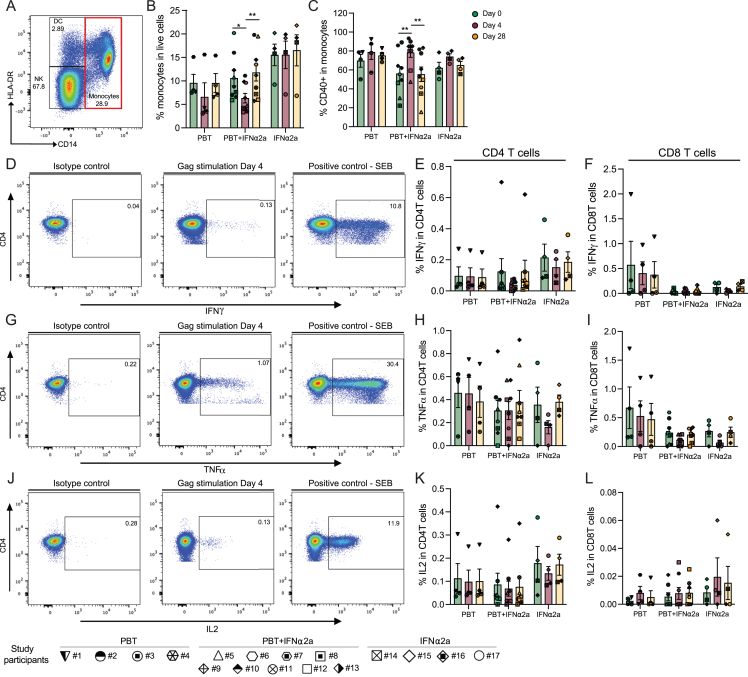
Analysis of monocytes and HIV-1-specific T cell responses during treatment with the study medication, related to Figure 3 (A) Representative flow cytometry dot plots indicating the gating strategy for defining monocytes. (B and C) Proportions of monocytes in live cells (B) and proportions of these cells expressing CD40 (C), determined at indicated time points in the study. (D–L) Representative flow cytometry dot plots and bar diagrams representing the expression of IFN-γ (D–F), TNF-α (G–I), or IL-2 (J–L) after stimulation with gag peptides in CD4^+^ T cells (E, H, and K) or CD8 T cells (F, I, and L); stimulation with SEB was used as a positive control. Vertical bars reflect mean and SEM.

We next conducted multiparametric flow cytometry assays to evaluate phenotypic changes in innate and adaptive immune cells during treatment with panobinostat and/or PEG-IFN-α2a. We noted that the frequencies of CD14^−^ CD11c^−^ CD123^+^ plasmacytoid dendritic cells (pDCs) did not change significantly during treatment with PEG-IFN-α2a, but the proportions of pDC subsets expressing the migratory marker CCR7 and the co-stimulatory molecules CD40 and CD86 increased in participants receiving PEG-IFN-α2a, either alone or in combination with panobinostat (Figures 3A and 3B). The fractions of CD14^−^ CD11c^+^ CD1c^+^ myeloid dendritic cells (mDCs type 2/3^40^) expressing the co-stimulatory molecules CD83, CD40, and CD86 also increased after PEG-IFN-α2a treatment in study arms B and C (Figure 3C). Similarly, a rise in the relative proportion of CD14^+^ HLA-DR^+^ monocytes expressing CD40 was noted during treatment with the combined study medication (Figures S2A–S2C). Proportions of CD16^+^ CD56^+^ natural killer (NK) cells, the main cytotoxic effector cell component of the innate immune system, also expanded during combined treatment, specifically the subsets expressing the activating markers CD38 and NKp30 (Figures 3D and 3E). Furthermore, the subgroups of T cells expressing granzyme A, granzyme B, or perforin increased in the combined treatment arm (Figures 3F–3I). The frequencies of HIV-1-specific T cells, determined by intracellular cytokine staining for IFN-γ, tumor necrosis factor alpha (TNF-α), or interleukin-2 (IL-2) following stimulation with an HIV-1 gag peptide pool, were unaffected by the study medication (Figures S2D–S2L). CD4^+^ T cell gene expression profiling, conducted by RNA sequencing (RNA-seq) at baseline, day 4, and day 28, revealed strong upregulation of a subset of IFN-stimulated genes (ISGs) in the majority of participants receiving PEG-IFN-α2a (Figures 3J and 3K), alone or in combination with panobinostat. Genes that were strongly upregulated following PEG-IFN-α2a treatment frequently encoded for proteins involved in innate immune recognition such as RIG-I-like receptor (RLR)/Toll-like receptor (TLR) family members (RIG-I, MDA5, LGP2, and TLR7) but also included other ISGs such as IFIT gene family members (IFIT1, IFIT2, and IFIT3), APOBEC3 genes (APOBEC3A and APOBEC3B), ISG15, MX1, OASL, OAS1, or SIGLEC-1.

### Evolution of genome-intact proviruses

**Figure 4:**
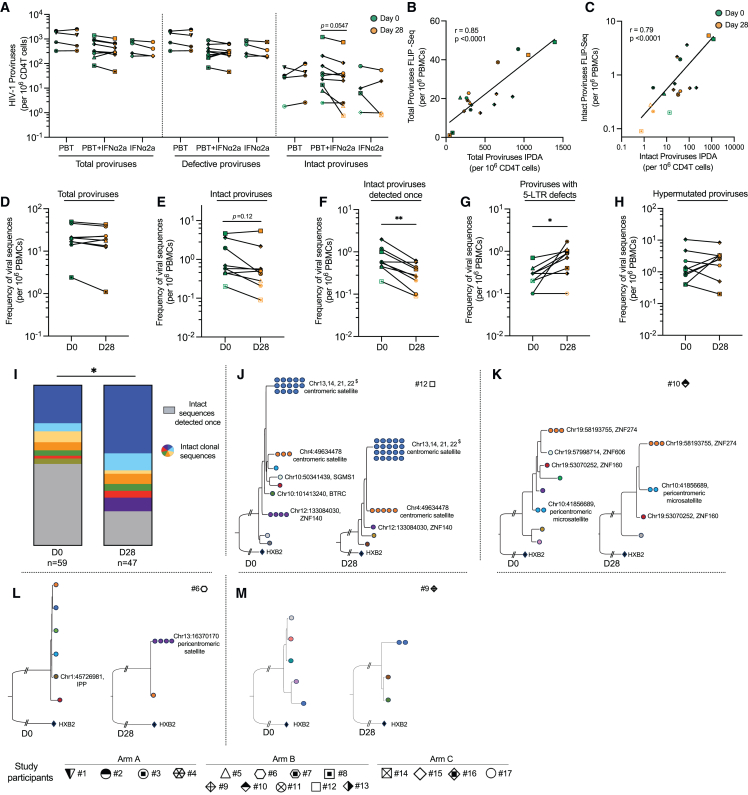
Evolution of intact and defective HIV-1 DNA during treatment with the study medication (A) Frequencies of total, defective, and intact proviruses per 10^6^ total CD4^+^ T cells as measured by the IPDA at days 0 and 28. Total proviruses were determined as the sum of intact, 5′ defective, and 3′ defective proviruses from each study participant. Empty symbols represent the limit of detection (LOD), defined as the total number of cells assayed without target identification. Note that due to the low frequencies of intact proviruses, data from total proviruses and defective proviruses are very similar. In study subject 12, a large proviral clone was detected that was classified as genome intact by IPDA but was defective when analyzed using FLIP-seq. (B and C) Spearman correlation between total (B) or intact (C) proviruses determined by FLIP-seq (and MIP-seq for participants #5, #6, #9, #10, and #12) and by IPDA. Note that FLIP-seq/MIP-seq results are reported as copies per million PBMCs, whereas IPDA data are reported as copies per million CD4^+^ T cells. (D–H) Frequencies of total HIV-1 proviruses (D), total intact proviruses (E), intact proviruses detected once at any of the analyzed time points (F), proviruses with 5-LTR defects (G), and hypermutated proviruses (H) per 10^6^ PBMCs, as measured by the FLIP-seq (and MIP-seq for participants #5, #6, #9, #10, and #12; ^∗^p < 0.05, Wilcoxon matched-pairs signed rank test). (I) Proportion of non-clonal intact sequences (detected once at any given time point, shown in gray) and clonal intact sequences (detected more than once at any given time point, colors denote members of the same clones) determined by FLIP-seq (and MIP-seq for participants #5, #6, #9, #10, and #12) at days 0 and 28. ^∗^p < 0.05, chi-squared test. (J and K) Maximum-likelihood phylogenetic trees for intact HIV-1 proviruses from study participants #6, #9, #10, and #12 obtained at days 0 and 28. Coordinates of chromosomal integration sites obtained by integration site loop amplification (ISLA) and corresponding gene names (where applicable) are indicated. Colors denote members of the same clone. $, integration sites that could not be definitively mapped to one exact genomic location due to positioning in repetitive centromeric satellite DNA present in multiple regions of the human genome. See also Figure S3.

**Figure S3:**
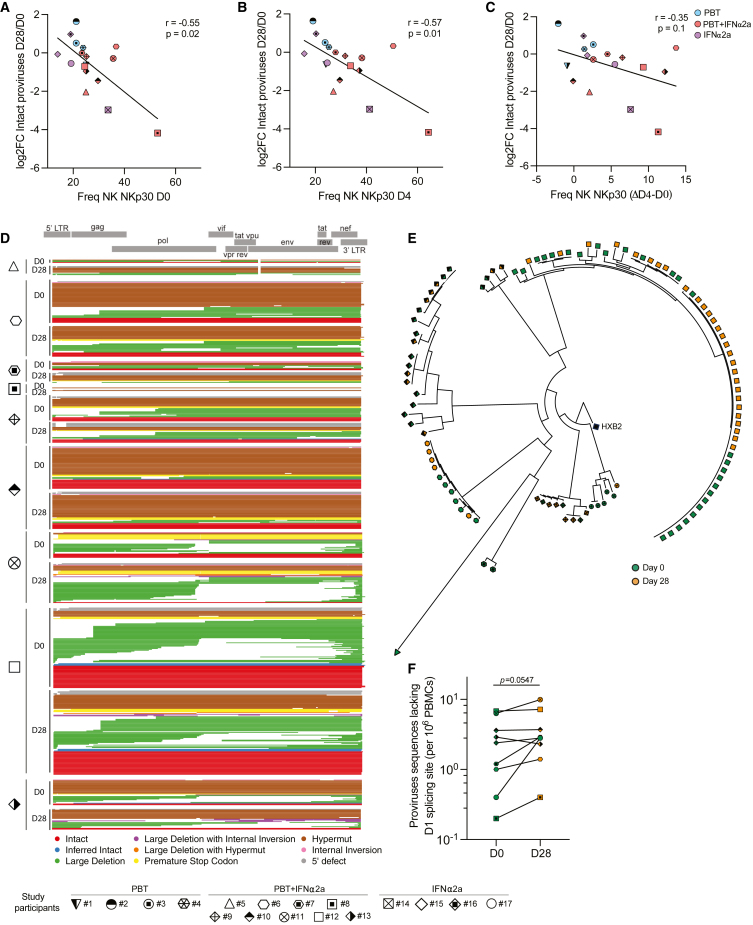
Molecular profiling of the proviral reservoir landscape during treatment with panobinostat and PEG-IFN-α2a, related to Figure 4 (A–C) Spearman correlation between the fold change (FC) in HIV-1 DNA levels (determined by IPDA) between days 28 and 0 (expressed as Log_2_FC) and the proportion of NK cells expressing NKp30 at day 0 (A), day 4 (B), and the difference (Δ) of NK cells expressing NKp30 between days 0 and 4 (C). (D) Virograms summarizing n = 614 individual HIV-1 proviral sequences from days 0 and 28 aligned to the HXB2 reference genome from each participant in study group B; color coding reflects the classification of proviral sequences. (E) Phylogenetic tree of all the intact sequences collected at days 0 and 28 by FLIP-seq and MIP-seq (n = 106) in study participants from group B. (F) Frequencies of proviruses with deletions in the D1 splice donor site at days 0 and 28 in study participants from study group B.

To evaluate changes in HIV-1 reservoir cells during treatment with the study medication, we used the intact proviral DNA assay (IPDA), which can identify HIV-1 genomes with a high probability to be genome-intact.^41^ We noted that the frequencies of defective proviruses did not change significantly in any of the study groups at any of the analyzed time points (Figure 4A). However, there was a trend for a reduction of intact HIV-1 proviruses in the combined treatment study group between days 0 and 28. Within these study participants, intact HIV-1 DNA declined by approximately 40% (mean of 179.04 vs*.* 108.42 intact HIV-1 DNA copies at day 0 vs. 28, p = 0.0547); this selective decrease is consistent with prior studies^42^^,^^43^^,^^44^^,^^45^ suggesting a higher immunological vulnerability of cells harboring intact proviruses, relative to those containing defective HIV-1 proviruses. Of note, a correlative analysis demonstrated that the proportions of NKp30^+^ CD56^+^ CD16^+^ NK cells at days 0 and 4 were statistically linked to reductions of intact proviruses during the study, possibly implying a functional role of innate effector cell immune responses in reducing viral reservoir cells (Figures S3A–S3C).

To complement these results, we used single-genome near full-length proviral sequencing (FLIP-seq)^5^^,^^6^ to profile changes in the proviral reservoir landscape during the combined treatment with panobinostat and PEG-IFN-α2a; in addition to distinguishing intact and defective proviral sequences, this approach permits to identify clones of sequence-identical proviruses that result from clonal proliferation of infected cells.^46^^,^^47^^,^^48^ For this purpose, we conducted a pair-wise analysis of similar numbers of PBMCs from days 0 and 28 in each study participant from group B, involving a total of 85.5 million PBMCs (44 million at day 0, 41.5 million at day 28). These studies identified a total number of n *=* 1,870 HIV-1 amplicons, n = 614 of which were selected for sequencing based on near full-length amplicon sizes during gel electrophoresis (Figure S3D); n = 106 of these sequences were classified as genome-intact (Figure S3E). Overall, we observed strong statistical associations between the numbers of total and genome-intact proviruses identified by IPDA (in purified CD4^+^ T cells) and by FLIP-seq (in total PBMCs) (Figures 4B and 4C); however, the absolute numbers of intact proviruses determined by FLIP-seq were smaller compared with IPDA results, likely reflecting the more limited efficacy of long-range viral DNA amplification^49^ and the fact that IPDA data are computationally adjusted for DNA shearing artifacts,^41^ whereas results from FLIP-seq are not. In one study participant (#12), we observed a large clone of proviral sequences (with a frequency of 5.1 viral copies/10^6^ PBMCs) that was classified as genome-intact by IPDA, although near full-length sequencing demonstrated lethal sequence deletions outside of the IPDA amplification regions.

Together, this detailed molecular analysis of the proviral landscape supported a trend for a decrease of intact proviruses in the combined treatment study arm (p = 0.12, Figure 4E); no major changes were observed for total HIV-1 proviruses (Figure 4D). Of note, we observed that the clonality of proviruses seemed to have a profound influence on the proviral reservoir dynamics: although the frequencies of intact proviruses detected only once at any of the analyzed time points decreased significantly during treatment with the combined study medication (mean of 0.77 vs. 0.33 intact DNA copies/million PBMCs, reduction of 58%, p = 0.004) (Figures 4F and 4I), we noted several large clones of intact proviruses that violated this pattern and persisted or expanded during the study, resulting in a diminished complexity of the post-treatment proviral landscape (Figure 4I) and in a numeric longitudinal increase of intact proviruses in “outlier” study participants (#12 and #6) (Figures 4J and 4L). A deeper analysis using matched integration site and proviral sequencing (MIP-seq)^12^ in study participants #12, #10, and #6 indicated that these persisting or expanding proviral clones were integrated in centromeric/peri-centromeric satellite DNA or zinc finger (ZNF) genes, both of which display repressive chromatin features and can confer a state of deeper latency associated with weaker susceptibility to latency-reversing agents,^13^^,^^50^^,^^51^ likely explaining why these proviruses appeared to be unaffected by the study medication. In study subject #9 (Figure 4M), we also observed one clone of intact proviruses that appeared to expand during treatment; however, we were unable to identify the corresponding proviral integration site of this low-abundance clone (with a frequency of approximately 1 copy in 5 million PBMCs) within the limited amounts of cells collected during the clinical trial. Of note, replication-incompetent proviruses with deletions in the 5′ LTR regions expanded during the study (Figure 4G); recent data suggest that such proviruses may be able to avoid host immune activity through more limited production of viral antigen involved in cytopathic effects and immune recognition,^52^ likely due to the deletion of the splicing donor site D1.^48^ In line with this observation, proviral sequences lacking D1 also tended to be selected for during the combined treatment, independently of alternative coinciding sequences defects (Figure S3F). No longitudinal changes were observed for proviral sequences with statistically significant hypermutations (Figure 4H). Taken together, these data suggest that intact proviruses can be vulnerable to the combined study medication; however, large clones of intact proviruses integrated in heterochromatin regions were able to persist and resist immune effects of the study drugs.

### Global changes in the proviral integration site landscape

**Figure 5:**
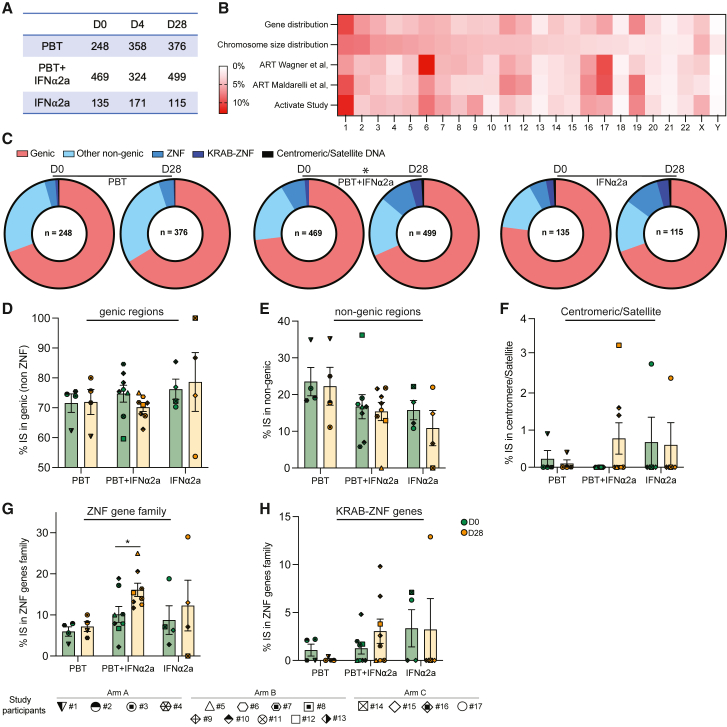
Changes in the proviral integration site landscape in the ACTIVATE study (A) Table describing the number of integration sites (IS) collected for each treatment arm at the indicated time points. (B) Heat map reflecting the contributions of individual chromosomes to the total human genome and to the numbers of detected HIV-1 integration sites from all study arms and time points. Data from Wagner et al.^53^ and from Maldarelli et al.^56^ are shown for comparison. (C) Pie charts showing the distributions of HIV-1 integration sites in genes, in non-genic regions, in the zinc-finger gene family, in KRAB-ZNF genes, and in centromeric/satellite DNA at days 0 and 28. The numbers within each pie chart indicate the total number of integration sites. (D–H) Proportions of integration sites located in genic regions (excluding ZNF genes) (D), non-genic regions (E), centromeric/satellite (F), and in the zinc-finger gene family (G) and KRAB-ZNF genes (H). The vertical bars reflect the mean with SEM,^∗^p < 0.05, Wilcoxon matched-pairs signed rank test or chi-squared tests. See also Figure S4.

The data described above, paired with prior investigations,^10^^,^^14^ raise the possibility that the immunological vulnerability of HIV-1 reservoir cells may depend on the proviral chromosomal integration sites; in particular, proviruses integrated in poorly accessible genomic regions with heterochromatin features may be less likely to respond to latency-reversing agents, leading to a selection advantage when host immune activity is therapeutically increased during shock and kill interventions. To evaluate global changes in the proviral chromosomal integration site landscape during treatment with the study medication, we characterized the chromosomal locations of 2,695 proviruses (n = 852 from baseline, n = 853 from day 4, and n = 990 from day 28) from bulk CD4^+^ T cells, using integration site loop amplification (ISLA)^53^ or, in a more limited number of proviruses, ligation-mediated PCR (LM-PCR)^54^^,^^55^ (Figure 5A). The median numbers of proviral integration sites collected from each study participant were 48, 44.5, and 49.5 at days 0, 4, and 28, respectively; in one out of the nine study subjects in arm B (participant #8) who had the lowest reservoir size among all study participants, experimental analysis of integration sites was unsuccessful.

**Figure S4:**
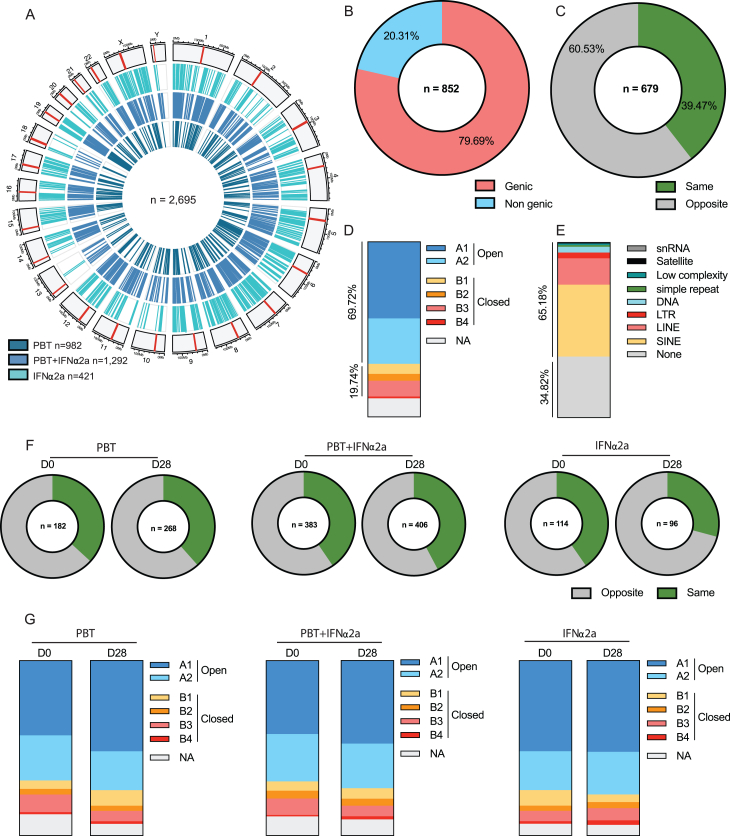
HIV-1 chromosomal integration site features in the ACTIVATE study, related to Figure 5 (A) Circos plot representing the genome-wide distribution of all HIV-1 integration sites (IS) identified in the three different treatment arms. The red bar in each chromosome represents the centromere. (B) Proportions of IS collected at day 0 (n = 852) integrated in genic or non-genic regions. (C) Proportions of IS collected at day 0 in genic regions (n = 679) integrated in the opposite or same direction as the host gene transcription. (D) Proportions of IS collected at day 0 mapped within chromatin structural compartments A and B and their respective sub-compartments as determined by Hi-C sequencing data.^58^ (E) Proportions of IS collected at day 0 in different repetitive genomic elements. (F) Proportions of IS in genic regions integrated in the opposite or same direction as host gene transcription collected at days 0 and 28. (G) Proportions of IS collected at days 0 and 28 mapped within chromatin structural compartments A and B and their respective sub-compartments.

To validate our dataset, we first studied features of integration sites obtained at baseline. Consistent with previous studies,^53^^,^^55^^,^^56^^,^^57^ we noted that proviruses tended to cluster in gene-rich chromosomes (e.g., chr1, chr17, and chr19) (Figure 5B; Figure S4A) and were predominantly located in genes (79.69% in total); 94.5% of genic integration sites were in introns, and 5.5% of genic integration sites were in exons (Figure S4B). Moreover, 60.5% of proviruses located in genes were integrated in opposite orientations to the host genes, 69.7% of proviruses aligned to regions in open three-dimensional (3D) chromatin compartments as determined by Hi-C reference data,^58^ and 65.18% were integrated in repetitive genomic elements such as long interspersed nuclear elements (LINEs) (15.06%) and short interspersed nuclear elements (SINEs) (41.06%) (Figures S4C–S4E); all of these characteristics are recognized and well-established features of the HIV-1 integration site landscape.^15^^,^^53^^,^^56^^,^^57^

During and after treatment with the study medication, we observed no significant changes in the proportions of proviruses located in genic vs. non-genic regions, in chromosomal orientations relative to host genes, and in the proportional distributions of proviral integration sites in 3D chromatin compartments (Figures 5D and 5E; Figures S4F and S4G). However, three study participants (#9, #10, and #12) who received the combined treatment regimen displayed increased proportions of integration sites in non-genic centromeric or satellite DNA at day 28, relative to baseline (Figure 5F); in two of these study persons (#10 and #12), an increase of clonal intact proviruses in centromeric satellite DNA was also observed during the study in our prior experiments focusing on the selective analysis of intact proviruses (Figures 4J and 4K). Moreover, the proportions of integration sites in ZNF gene family members significantly increased during treatment with the combined study medication (p = 0.032); a trend for an increase in the proportions of integration sites in Krüppel-associated box (KRAB)-domain-containing ZNF genes was also observed (Figures 5C, 5G, and 5H). As discussed above, centromeric satellite DNA and ZNF genes both display heterochromatin features enriched with repressive epigenetic histone modifications^59^; proviruses in such regions may be in a deeper state of latency and exhibit a more limited response to reactivation signals.^13^^,^^51^^,^^60^^,^^61^ Relative increases of proviruses in centromeres or satellite DNA and in ZNF family genes during treatment with panobinostat and PEG-IFN-α2a may reflect preferential immune-mediated elimination of HIV-1 sequences in more accessible chromatin regions and a reciprocal accumulation of proviruses integrated in chromosomal regions conferring transcriptional repression and higher resistance to latency-reversing agents.

### Proviral chromosomal locations relative to epigenetic features

**Figure 6:**
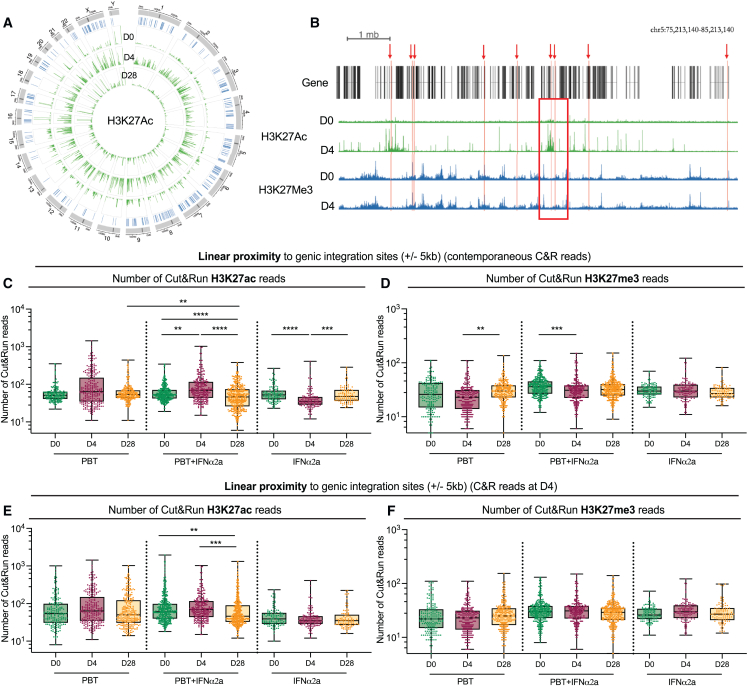
Longitudinal evolution of proviral chromosomal locations relative to epigenetic histone modifications in linear proximity (A) Circos plot representing the distribution of HIV-1 integration sites in the human genome (in blue) and the genome-wide H3K27ac peaks (in green) detected at days 0, 4, and 28 in the ACTIVATE study. (B) Representative genome browser snapshot reflecting the epigenetic environment surrounding HIV-1 integration sites. Integration sites (red arrows) were aligned to H3K27ac (in green) and H3K27me3 (in blue) Cut & Run seq data (C&R) from autologous isolated CD4^+^ T cells; the number of Cut & Run seq reads was calculated in ±5 kb intervals of each IS (example indicated by a red square). (C–F) Box and whisker plots showing the median, the 25th and 75th percentiles, and the maximum/minimum values of the read numbers of H3K27ac (C and E) or H3K27me3 (D and F). Data reflect Cut & Run seq reads in linear proximity to IS in genic regions at indicated study time points using the Cut & Run seq data from autologous CD4^+^ T cells from contemporaneous study time points (C and D) or using the autologous Cut & Run seq data collected at day 4 (E and F). ^∗^p < 0.05, ^∗∗^p < 0.01, ^∗∗∗^p < 0.001, ^∗∗∗∗^p < 0.0001, Kruskall-Wallis test, false discovery rate (FDR)-adjusted p values are shown. See also Figure S5.

**Figure S5:**
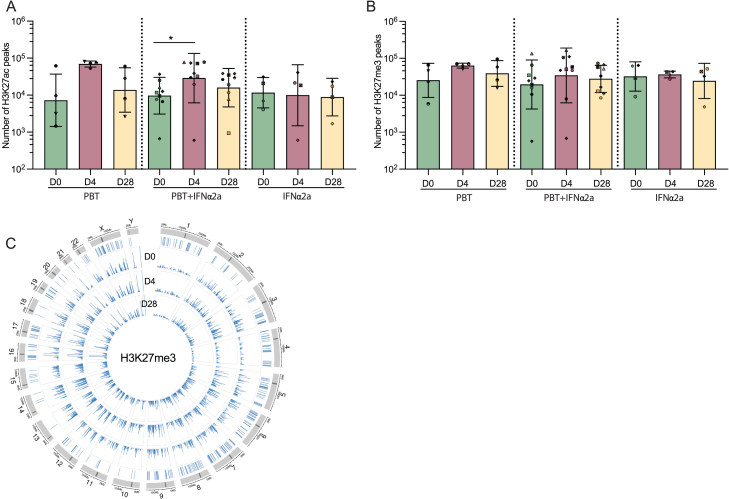
Genome-wide analysis of epigenetic histone modifications in CD4^+^ T cells, related to Figures 6 and 7 (A and B) Bar diagrams representing the numbers of H3K27ac (A) or H3K27me3 (B) Cut & Run seq peaks detected at day 0, 4, or 28 (^∗^p < 0.05, Friedman test followed by Dunn’s multiple comparisons). Bars indicate mean with SEM. (C) Circos plot representing the distribution of IS within the genome and the H3K27me3 peak detected at days 0, 4, and 28 after panobinostat treatment.

In a subsequent analysis, we tested the hypothesis that proviral integration site locations relative to H3K27ac marks, the molecular target sites for panobinostat, may more broadly affect persistence and selection of proviruses during treatment with the study medication; in particular, proviral proximity to H3K27ac marks might increase the vulnerability to panobinostat, whereas enhanced distance to H3K27ac marks might confer protection from panobinostat-mediated reactivation and subsequent immune recognition. To address this, we conducted a genome-wide assessment of H3K27ac histone marks, using cleavage under targets and release under nuclease sequencing (Cut & Run seq)^62^ to identify chromatin regions enriched with H3K27ac marks; for control purposes, we similarly analyzed genomic DNA bound to the inhibitory chromatin mark H3K27me3. We performed these assays on autologous isolated CD4^+^ T cells from three time points (days 0, 4, and 28) in all study participants. For individuals who received panobinostat, alone or in combination with PEG-IFN-α2a, a profound global genome-wide increase in H3K27ac marks was observed on day 4, as expected; by day 28, global H3K27ac marks had normalized to baseline levels (Figure 6A; Figure S5A). By contrast, longitudinal changes in H3K27me3 marks during treatment with the study medication were less pronounced (Figures S5B and S5C).

To study the epigenetic chromatin features around each proviral integration site, we aligned proviral chromosomal coordinates from each time point to the corresponding autologous Cut & Run seq data and calculated the number of reads in linear proximity (±5 kb) to each genic integration site (Figure 6B); our prior work suggested that epigenetic signals surrounding integration sites within 5 kb intervals can influence proviral transcriptional activity.^13^ We observed markedly increased H3K27ac-specific Cut & Run seq reads surrounding the integration sites on day 4 for participants who received panobinostat, alone or in combination, consistent with the global increase in H3K27ac marks after panobinostat treatment (Figure 6C). Of note, there were fewer H3K27ac reads in proximity of genic proviral integration sites on day 28, compared with baseline, in the majority of participants receiving the combined study medication; no such change was seen in individuals receiving panobinostat alone (Figure 6C). In participants who received PEG-IFN-α2a only, a transient decrease of H3K27ac reads in linear proximity to proviruses occurred at day 4 but was not detectable at day 28; the reason for this transient decline is currently unknown and requires further evaluation in future studies. As an additional analysis step, we aligned genic integration site coordinates from all three time points (days 0, 4, and 28) to H3K27ac Cut & Run seq data from day 4, considering that epigenetic data from this time point reflect the totality of chromatin regions with constitutive and panobinostat-inducible H3K27ac marks. The frequencies of these H3K27ac reads surrounding proviral integration sites isolated at the three time points were similar for study persons who received panobinostat only or PEG-IFN-α2a only (Figure 6E). However, for participants who received the combined treatment, integration sites from day 28 were surrounded by markedly fewer H3K27ac reads compared with baseline in this analysis (Figure 6E). No systematic longitudinal changes were observed for H3K27me3 reads surrounding proviral integration sites when aligning integration site coordinates from days 0, 4, and 28 to autologous H3K27me3 Cut and Run seq data from their corresponding contemporaneous time points (Figure 6D) or from day 4 only (Figure 6F). Together, these results suggest preferential elimination of proviruses in close proximity to H3K27ac-loaded chromatin in recipients of the combined treatment regimen, presumably due to the higher propensity of these proviruses to respond to panobinostat-induced reactivation signals.

**Figure 7:**
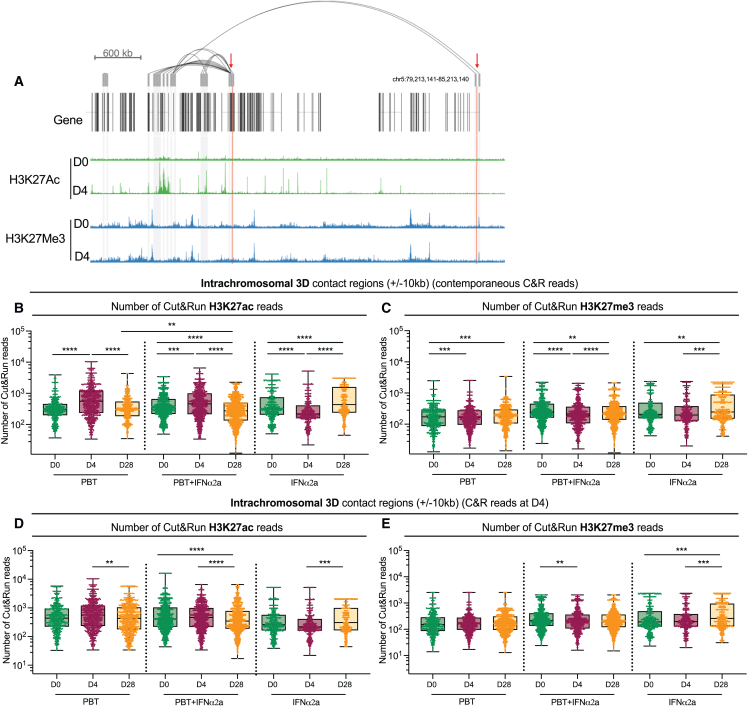
Longitudinal evolution of proviral chromosomal locations relative to epigenetic features in three-dimensional chromatin contact regions (A) Genome browser snapshot highlighting intra-chromosomal contact regions of HIV-1 integration sites highlighted by red arrows. Alignments of Cut & Run seq data for H3K27ac and H3K27me3 are also visualized. (B–E) Box and whisker plots showing the median, the 25th and 75th percentiles, and the maximum/minimum values of the read numbers of H3K27ac (B and D) or H3K27me3 (C and E) Cut & Run seq (C&R) reads in intrachromosomal 3D contact regions to genic integration sites (±10 kb) detected at indicated study time points. 3D contact regions of integration sites were identified using Hi-C data from primary CD4^+^ T cells at 20kb binning resolution. Autologous Cut & Run seq data from contemporaneous study time points (B and C) or from day 4 (D and E) are shown. ^∗^p < 0.05, ^∗∗^p < 0.01, ^∗∗∗^p < 0.001, ^∗∗∗∗^p < 0.0001, Kruskall-Wallis test, FDR-adjusted p values are shown. See also Figure S5.

Since proviral responsiveness to panobinostat may also be influenced by H3K27ac marks in 3D chromatin contact regions of HIV-1 proviruses, we aligned genic chromosomal integration site coordinates to *in situ* Hi-C data previously collected from CD4^+^ T cells from ART-treated persons,^13^ allowing us to identify intrachromosomal chromatin regions likely to interact with proviral integration sites through 3D contacts (Figures 7A–7E). This analysis demonstrated a significant decrease in the numbers of H3K27ac Cut & Run seq reads in proviral 3D contact regions on day 28, relative to day 0; this effect was selectively observed among participants who received the combined treatment and was noted when integration site coordinates were aligned to 3D H3K27ac reads from the respective contemporaneous time points (Figure 7B) or to 3D H3K27ac reads from day 4 only (reflecting the totality of constitutive and panobinostat-inducible H3K27ac reads) (Figure 7D). Some differences among H3K27me3 reads in 3D contact regions of HIV-1 integration sites from days 0, 4, and 28 also reached statistical significance in these large datasets; however, median fold changes in these comparisons were small and unlikely to be of biological relevance (Figures 7C and 7E). Collectively, these observations suggest that proviruses surviving the combined treatment are preferentially located in chromosomal regions with fewer H3K27ac marks in their linear and 3D proximity.

## Discussion

Although HIV-1 persists life-long in individuals undergoing antiretroviral therapy, growing evidence suggests that the human immune system can engage HIV-1 reservoir cells.^14^^,^^63^ Proviruses with detectable residual transcriptional activity, frequently integrated in immediate proximity to activating epigenetic chromatin marks, appear to be more vulnerable to such host immune activities, most likely because viral gene expression increases the visibility of infected cells to immune recognition. Such a view implies that the pharmacological induction of proviral gene expression may facilitate immune clearance of HIV-1 reservoir cells and represents the premise of shock-and-kill interventions designed to reduce HIV-1 persistence. In this study, we show that the HDACi panobinostat co-administered with an innate immune modulator can increase proviral gene transcription and induce a structural transformation of the HIV-1 reservoir cell pool characterized by a disproportionate overrepresentation of HIV-1 proviruses integrated in ZNF genes, in chromatin regions with reduced H3K27ac marks, and, to a lesser extent, in centromeric/satellite DNA regions. Moreover, we observed a numeric decrease of non-clonal intact proviruses, an overall reduction in the structural complexity and phylogenetic composition of the landscape of intact proviruses, and an enhanced number of proviruses with 5-LTR deletions; these changes are reminiscent of immune selection mechanisms in the reservoir cell pool that occur naturally during long-term ART^12^^,^^14^^,^^45^^,^^52^^,^^55^ and may be accelerated and intensified through the study medication tested in the described clinical study. Although effect sizes and responsiveness to the study medication varied among the study participants, these results from a small, exploratory human clinical trial suggest that HIV-1 proviruses are vulnerable and can be sensitized to immune clearance, at least when integrated near activating chromatin marks targeted by HDACi. Collectively, we propose that shock-and-kill interventions can accelerate and amplify naturally occurring immune selection forces that promote the longitudinal accumulation of HIV-1 proviruses in transcriptionally repressed chromatin locations.^14^

Although induction of HIV-1/SIV transcription through pharmacological latency-reversing agents has previously been described in human^22^^,^^23^^,^^24^^,^^64^^,^^65^ and animal studies,^66^^,^^67^ their effects on the viral reservoir profile have been difficult to assess in the past. In our work, we performed a detailed longitudinal analysis of proviral integration sites, permitting us to evaluate qualitative changes in the viral integration site landscape relative to epigenetic features targeted by panobinostat; such an analytic approach is based on the hypothesis that the susceptibility to latency-reversing agents may vary profoundly among individual proviruses and is likely modulated by chromatin characteristics surrounding the proviral genomic locations.^13^^,^^68^^,^^69^ In this analysis, chromatin regions with higher H3K27ac loads displayed evidence for more effective purging of HIV-1 proviruses during treatment with panobinostat and PEG-IFN-α2a, whereas proviruses surviving treatment with the combined study medication seemed to be preferentially integrated in chromatin segments with limited H3K27ac marks in linear and 3D proximity. This finding suggests that chromatin with reduced H3K27ac marks may represent a “safe haven” and a possible “sanctuary site” for HIV-1 proviruses, due to a chromatin microenvironment that protects HIV-1 proviruses from panobinostat-induced reactivation signals. However, we acknowledge that this analysis included total (intact and defective) proviruses and that susceptibility to latency-reversing effects may have been influenced both by chromosomal location and by proviral sequence characteristics. Our analysis also shows a selection of HIV-1 proviruses in the ZNF gene family during the combined treatment. Enrichment of intact proviruses in ZNF genes has been described in several studies,^7^^,^^10^^,^^12^^,^^15^^,^^70^^,^^71^ suggesting that integration in these genes can support persistence of HIV-1 DNA and may immunize proviruses against host immune attacks. The precise mechanisms promoting persistence of proviruses in ZNF genes may involve proviral transcriptional repression by the human silencing hub (HUSH) complex,^72^^,^^73^ but this remains an area of active investigation.

In comparison to qualitative changes in proviral chromatin positioning relative to epigenetic chromatin features, quantitative changes in HIV-1 reservoir cells were more difficult to evaluate. Using the IPDA, we noted a trend for reduction of intact proviruses, without demonstrable effect on defective proviruses; however, we emphasize that these data likely reflect net effects of underlying changes in the clonal compositions of HIV-1 reservoir cells, with persistence and expansion of some clones and extinction of others. A more detailed analysis of the proviral landscape using single-genome near full-length next-generation sequencing indeed demonstrated that in some of the study participants, large clones of intact proviruses integrated in heterochromatin locations, specifically in satellite DNA, persisted or expanded; presumably, cells harboring these clones are less responsive to latency-reversing effects of panobinostat, translating into a longitudinal selection advantage and, possibly, into PEG-IFN-α2a-driven clonal proliferation unimpaired by proviral reactivation and immune-mediated killing. By contrast, intact proviruses detected only once were selected against during treatment with the combined study medication. Unfortunately, at the current stage of technology development, an analysis involving MIP-seq^12^ cannot be comprehensively conducted for larger numbers of longitudinal samples collected from clinical trial participants; moreover, the limited amounts of cells that could be safely collected by repetitive sampling during the short period of the study did not permit us to expand the analysis of integration sites of intact proviruses.

The type of immune responses that are most effective in driving selection HIV-1 reservoir cells is unclear at present. In this study, an innate immune modulator was chosen to enhance immune effects against viral reservoir cells; this approach was complementary to alternative studies in which HDACis were combined with therapeutic vaccines^25^^,^^26^ or with broadly neutralizing antibodies^27^^,^^28^ and was supported by a series of prior studies suggesting associations between innate immune activity and viral reservoir cell evolution.^16^^,^^18^^,^^19^^,^^20^^,^^32^ In theory, innate immune mechanisms may be able to target viral reservoir cells through cell-intrinsic recognition of viral transcripts by cytoplasmic^74^ or endosomal immune sensors.^75^ Although the transcripts induced during treatment with panobinostat frequently lacked poly-A tails and functional rev response elements (RREs), they may be able to access cytoplasmic immune sensors following nuclear export via a rev-independent pathway^76^^,^^77^; cell-intrinsic immune recognition of HIV-1 transcripts has indeed been described in prior work.^74^^,^^78^ Moreover, viral reservoir cells may be targeted by innate immune effector cells that might identify virally infected cells through as-of-yet unidentified immune receptors. In support of this assumption, we observed an expansion of cytotoxic NK cells and activated mDC in our study and an inverse association between levels of intact proviruses and proportions of NKp30^+^ NK cells. The recent advent of single-cell assays^79^^,^^80^^,^^81^^,^^82^^,^^83^ that can capture the phenotype or the transcriptome of virally infected cells in an unperturbed state may help to better evaluate the susceptibility of HIV-1 reservoir cells to innate immune responses in future clinical studies, including those involving pharmacological increases of proviral transcription. Notably, we did not notice any major influence of the study medication on HIV-1-specific T cell responses, even though IFN-α2a can drive and expand antigen-specific T cells^84^ and panobinostat-induced p24 protein production resulted in an expansion of HIV-1-specific T cell responses *in vitro*.^39^

In sum, our study shows that the administration of as few as three doses of panobinostat, combined with a single dose of PEG-IFN-α2a, can lead to a marked change in the structure and composition of the HIV-1 reservoir cell pool. In our opinion, the results presented here suggest that future shock-and-kill studies, ideally involving optimized and more potent latency-reversing agents that can be repetitively dosed over longer periods of time, are warranted; such future studies might possibly support the evolution of a reservoir profile dominated by proviruses integrated in heterochromatin positions, imitating the integration site profile of endogenous retroviruses in the human genome^85^ and of intact HIV-1 proviruses in elite controllers^10^ and post-treatment controllers.^14^ In addition, we argue that future insight into the specific immune mechanism able to target HIV-1-infected cells after disruption of viral latency will be critical for increasing the efficacy of shock-and-kill interventions.

### Limitations of the study

The results of this study are, to our knowledge, the first data to demonstrate an effect of shock-and-kill interventions on qualitative features of the HIV-1 reservoir profile; however, we do not claim that these effects are likely to reach clinical significance and for this reason did not conduct an analytical treatment interruption in this study. We acknowledge that the number of study participants in our study was small, that most of our study persons were male, that effect sizes were moderate and varied among individual study subjects, and that a 1-week treatment with a latency-reversing agent is likely insufficient for reactivating a clinically relevant number of proviruses. Furthermore, despite the use of two different methods to optimize HIV-1 integration site analysis, the total number of integration events remained limited; future technological advances may permit profiling a much larger number of integration site coordinates. An additional notable limitation of this study is our inability to evaluate viral reservoir changes in lymphoid tissues, where the majority of reservoir cell reside^86^; however, serial tissue sampling in study participants was considered too invasive and risky in this exploratory clinical trial. Finally, a combined assessment of proviral integration sites and histone modifications in single participant-derived cells is beyond of what current single-cell technologies are able to achieve; therefore, our study relied on aligning proviral integration site coordinates to H3K27ac signals in DNA from bulk CD4^+^ T cells.

## STAR★Methods

### Key resources table

**Table:** 

REAGENT or RESOURCE	SOURCE	IDENTIFIER
**Antibodies**		

Anti-CD95 PE/Dazzle 594	BioLegend	RRID: AB_2564221
Anti-CD3 Alexa Fluor 700	BioLegend	RRID: AB_493741
Anti-CCR7 Alexa Fluor 647	BioLegend	RRID: AB_10917385
Anti-CD45RA Brillant Violet 711	BioLegend	RRID: AB_2563815
Anti-CD8a Brillant Violet 510	BioLegend	RRID: AB_2561942
Anti-CD4 BUV395	BD Biosciences	RRID: AB_2738917
Anti-NKp30 PerCP/Cyanine 5.5	BioLegend	RRID: AB_2716094
Anti-NKG2A (CD159a) FITC	Miltenyi	RRID: AB_2655382
Anti-NKp46 PE/Cyanine 7	BioLegend	RRID: AB_2561620
Anti-NKG2D PE/Dazzle 594	BioLegend	RRID: AB_2687172
Anti-Siglec7 (CD328) PE	Miltenyi	RRID: AB_2657533
Anti-CD3 APC/Cyanine 7	BioLegend	RRID: AB_314053
Anti-CD14 APC/Cyanine 7	BioLegend	RRID: AB_830693
Anti-CD19 APC/Cyanine 7	BioLegend	RRID: AB_314247
Anti-CD38 Alexa Fluor 700	BioLegend	RRID: AB_2072781
Anti-NKG2C (CD159c) APC	R&D Systems	Cat#FAB138A-100 (clone 134591)
Anti-PD1 Brillant Violet 785	BioLegend	RRID: AB_2563680
Anti-CD56 Brillant Violet 711	BioLegend	RRID: AB_2562417
Anti-CD161 BV650	BD Biosciences	RRID: AB_2738456
Anti-CD57 Pacific Blue	BioLegend	RRID: AB_2063197
Anti-CD16 BUV395	BD Biosciences	RRID: AB_2744293
Anti-CD3 PerCP/Cyanine 5.5	BioLegend	RRID: AB_1575008
Anti-CD19 PerCP/Cyanine 5.5	BioLegend	RRID: AB_2073119
Anti-CD20 PerCP/Cyanine 5.5	BioLegend	RRID: AB_893285
Anti-CD56 PerCP/Cyanine 5.5	BioLegend	RRID: AB_893389
Anti-CD40 FITC	BioLegend	RRID: AB_1186034
Anti-CD1c PE/Cyanine 7	BioLegend	RRID: AB_1953227
Anti-ICOSL (CD275) PE-CF594	BD Biosciences	RRID: AB_2738726
Anti-HLA-DR PE	R&D Systems	Cat#FAB4869P-100 (clone L203)
Anti-CD83 APC/Cyanine 7	BioLegend	RRID: AB_2566392
Anti-CD14 Alexa Fluor 700	BioLegend	RRID: AB_2566716
Anti-CD141 (BDCA-3) APC	BD Biosciences	RRID: AB_2738608
Anti-CCR7 Brillant Violet 785	BioLegend	RRID: AB_2563630
Anti-PD-L1 Brillant Violet 711	BioLegend	RRID: AB_2565764
Anti-CD86 Brillant Violet 650	BioLegend	RRID: AB_11126752
Anti-CD123 Brillant Violet 510	BioLegend	RRID: AB_2562067
Anti-CD11c Brillant Violet 421	BioLegend	RRID: AB_2564484
Anti-CD16 BUV395	BD Biosciences	RRID: AB_2744293
Anti-PD1 PerCP/Cyanine 5.5	BioLegend	RRID: AB_1595561
Anti-CD3 PE/Cyanine 7	BioLegend	RRID: AB_439781
Anti-CD8a APC/Cyanine 7	BioLegend	RRID: AB_314134
Anti-CD4 BUV395	BD Biosciences	RRID: AB_2738917
Anti-Perforin FITC	BioLegend	RRID: AB_2571967
Anti-Eomes PE-eFluor	eBioscience	Cat#61-4877-42 (clone WD1928)
Anti-T-bet PE	BioLegend	RRID: AB_2028583
Anti-Granzyme A Alexa Fluor 700	BioLegend	RRID: AB_961343
Anti-IFNɤ Alexa Fluor 647	BioLegend	RRID: AB_493031
Anti-IL-2 Brillant Violet 650	BioLegend	RRID: AB_11147166
Anti-Granzyme B BV510	BD Biosciences	RRID: AB_2738174
Anti-TNFα Brillant Violet 421	BioLegend	RRID: AB_10960738
Anti-H3K27ac	Cell Signaling Technology	RRID: AB_10949503
Anti-H3K27me3	Cell Signaling Technology	RRID: AB_2616029
Anti-rabbit IgG	Antibodies-online	RRID: AB_10775589
anti-acetyl histone H3	Millipore	RRID: AB_2115283
Goat Anti-Rabbit IgG (H+L) Antibody, FITC conjugate	Millipore	RRID: AB_390182
Co-Stimulatory Antibodies (CD28/CD49d)	BD Biosciences	RRID: AB_647457
Anti-Human CD107a BV786	BD Biosciences	RRID: AB_2738458
Anti-Human CD107b BV786	BD Biosciences	RRID: AB_2739170
Anti-Human CD107b BV786	BD Biosciences	RRID: AB_2739170

**Biological samples**		

PBMC samples from ACTIVATE study participants	Massachusetts General Hospital	N/A

**Chemicals, peptides, and recombinant proteins**		

Concanavalin-A coated magnetic beads	Bangs Laboratory	Cat#BP531
Digitonin	Millipore	Cat#300410
CUTANA™ pAG-MNase for ChIC/CUT&RUN 50 Rxns	Epicypher	Cat#15-1016
LIVE/DEAD™ Fixable Blue Dead Cell Stain Kit	Thermo Fisher Scientific	Cat#L23105
FOXP3 / Transcription factor staining buffer set	Invitrogen / eBioscience	Cat#00-5523-00
FcR Blocking Reagent, human	Miltenyi	Cat#130-059-901
Paraformaldehyde solution 4% in PBS	Thermo Fisher Scientific	Cat#J19943.K2
Distilled, deionized or RNAse-free H2O	Promega	Cat#P1197
Manganese Chloride (MnCl2)	Sigma Aldrich	Cat#203734-5g
Calcium Chloride (CaCl2)	Fisher Scientific	Cat#BP510-100g
Potassium Chloride (KCl)	Sigma Aldrich	Cat#P3911-25g
Hydroxyethyl piperazineethanesulfonic acid	Sigma Aldrich	Cat#H3375-25g
Sodium chloride (NaCl)	Sigma Aldrich	Cat#S5150-1L
Ethylenediaminetetraacetic acid (EDTA)	Sigma Aldrich	Cat#E7889
Ethylene glycol-bis(β-aminoethyl ether)-N,N,N,N-tetraacetic acid (EGTA)	Sigma Aldrich	Cat#E3889-10G
Roche Complete Protease Inhibitor EDTA-Free tablets	Sigma Aldrich	Cat#11873580001
RNase A, DNase and protease-free (10 mg/ml)	Thermo Fisher Scientific	Cat#EN0531
Agencourt AMPure XP magnetic beads	Beckman Coulter	Cat#A63882
Sodium dodecyl sulfate (SDS)	Sigma Aldrich	Cat#L4509-25G
Proteinase K	Thermo Fisher Scientific	Cat#EO0491
Phenol-chloroform-isoamyl alcohol	Thermo Fisher Scientific	Cat#15593031
Chloroform	Sigma Aldrich	Cat#366919-1L
1 M Tris-HCl pH 8.0	Fisher Scientific	Cat#AAJ22638AP
20 mg/ml Glycogen	Sigma Aldrich	Cat#10901393001
Agencourt RNAClean XP	Beckman Coulter	Cat#A63987
SUPERase⋅ In™ RNase Inhibitor (20 U/μL)	Life Technologies	Cat#AM2696
SuperScript™ III Reverse Transcriptase	Life Technologies	Cat#18080085
dNTP Mix (10 mM ea)	Life Technologies	Cat#18427088
Ethanol 200 Proof	Decon Labs	Cat#2716
Triton X-100	Millipore	Cat#648463
Peptide Pool, Human Immunodeficiency Virus Type 1 Subtype B (Consensus) Gag Region	NIH AIDS Reagent Program	Cat#ARP-12425
Normal Rabbit Serum	Millipore	Cat#NS01L
Enterotoxin Type B, Staphylococcus aureus (SEB)	Sigma-Aldrich	Cat#324798
Brefeldin A	BioLegend	Cat#420601
Monensin	BD Biosciences	Cat#554724
DNase I	Invitrogen	Cat#18068015

**Critical commercial assays**		

NEBNext Ultra II DNA Library prep Kit	New England Biolabs	Cat#E7645S
NEBNext® Multiplex Oligos for Illumina® (Dual Index Primers Set 1)	New England Biolabs	Cat#E7600S
NextSeq 500/550 High Output v2.5 kit	Illumina	Cat#20024906
Nextera XT DNA Sample Preparation Kit	Illumina	Cat#FC-131-1096
Nextera XT Index kit	Illumina	Cat#FC-131-1002
EasySep Human CD4^+^ T cell isolation Kit	StemCell	Cat#17952
Dynabeads CD4 positive isolation kit	Invitrogen	Cat#11145D
Qubit 1X dsDNA HS Assay Kit	Invitrogen	Cat#Q33231
High Sensitivity D1000 Reagents	Agilent Technologies	Cat#5067-5585
ddPCR Supermix for Probes (No dUTP)	Bio-Rad	Cat#1863024
REPLI-g Single Cell Kit	Qiagen	Cat#150345
Invitrogen Platinum Taq DNA Polymerase High Fidelity	ThermoFisher Scientific	Cat#11304102

**Deposited data**		

Cut&Run sequencing data	This paper	GEO: GSE234625
RNA sequencing data	This paper	GEO: GSE234625
Hi-C data from CD4^+^ T cells	Einkauf et al.^13^	GEO: GSE168337

**Oligonucleotides**		

All the primers used have been described in Lee et al.^5^ and Einkauf et al.^12^	Millipore Sigma/IDT/Qiagen	N/A

**Software and algorithms**		

BD FACS Diva	BD Biosciences	N/A
MACS2	Zhang et al.^87^	N/A
DeepTools	Ramirez et al.^88^	N/A
Diffbind	Ross-Innes et al.^89^	N/A
DESeq2	Love et al.^90^	N/A
Ingenuity Pathway Analysis	Qiagen	version 90348151
FlowJo	Tree Star LLC	version 10.5.3
Wave	Agilent Technologies	version 2.6.0
QuantaSoft software	Bio-Rad	Cat#1864011
GraphPad	Prism	version 9.5.1
MUSCLE	Edgar^91^	http://www.drive5.com/muscle/
Automated in-house proviral intactness bioinformatic pipeline in Python	Lee et al.^5^	https://github.com/BWH-Lichterfeld-Lab/Intactness-Pipeline
Los Alamos National Laboratory (LANL) HIV Sequence Database Hypermut 2.0	Rose and Korber^92^	https://www.hiv.lanl.gov/content/sequence/HYPERMUT/background.html
Geneious Prime 2023.2.1	Biomatters	https://www.geneious.com/download/
Prism	Graphpad, https://www.graphpad.com/scientific-software/prism	Version 10.0.2 (171)
R	R Core Team and R Foundation for Statistical Computing, https://www.r-project.org	version 4.1.1
RepeatMasker	Institute for Systems Biology	http://www.repeatmasker.org/
FastQC (v0.11.9)	Babraham Bioinformatics	https://www.bioinformatics.babraham.ac.uk
bwa-mem	Li and Durbin^93^	http://maq.sourceforge.net/
Ensembl (V109)	Ensembl	www.ensembl.org
UCSC Genome Browser	UCSC	www.genome.ucsc.edu
GENCODE (V43)	GENCODE	www.gencodegenes.org
RSEM (v1.2.22)	Li and Dewey^94^	http://deweylab.github.io/RSEM/
STAR aligner software (2.5.1b)	ENCODE	https://www.encodeproject.org/software/star/
DAVID v6.8	Huang da et al.^95^	https://david.ncifcrf.gov/
FitHiC2	https://bioconductor.org/packages/release/bioc/html/FitHiC.html	version 1.20.0
DESeq2	Love et al.^90^	https://bioconductor.org/packages/release/bioc/html/DESeq2.html

**Other**		

Biorender	N/A	https://biorender.com
NextSeq 500 Instrument	Illumina	https://www.illumina.com/systems/sequencing-platforms/nextseq.html
QX200 Droplet Digital PCR System	Bio-Rad	https://www.bio-rad.com/en-us/life-science/digital-pcr/qx200-droplet-digital-pcr-system
96-well T100 PCR thermal cycler	Bio-Rad	1861096
Illumina MiSeq performed by MGH CCIB DNA Core facility	Illumina/MGH CCIB DNA Core	https://dnacore.mgh.harvard.edu/

### Resource availability

#### Lead contact

Further information and requests for resources and reagents should be directed to and will be fulfilled by the lead contact, Mathias Lichterfeld (mlichterfeld@mgh.harvard.edu).

#### Materials availability

This study did not generate new unique reagents.

#### Data and code availability

• This paper does not report original code.
• RNA-Seq and Cut&Run-seq datas were deposited to Gene Expression Omnibus (GEO) with the following accession numbers: GSE234625.
• Proviral integration sites are listed in Table S7.
• Proviral sequences: Due to study participant confidentiality concerns, viral sequencing data cannot be publicly released but will be made available to investigators upon reasonable request and after signing a data sharing agreement.
• Any additional information required to reanalyze the data reported in this paper is available from the lead contact upon reasonable request.

### Experimental model and study participant details

#### Study design

The ACTIVATE study was a prospective, open-label, randomized, dose-escalation exploratory pilot clinical trial involving people living with HIV-1 treated with suppressive combination antiretroviral therapy, conducted as a single-site study at Massachusetts General Hospital in Boston, MA. The study was approved by the local IRB (protocol number 2015P000858) and was conducted under US FDA IND 123446 and NIAID DAIDS-ES ID 12049; the study was registered at https://clinicaltrials.gov under NCT02471430.

#### Participants

Individuals aged 18-65 years living with HIV-1 who have received antiretroviral therapy with undetectable viral loads for at least two years and a CD4^+^ T cell count >400 cells/mm^3^ were invited to participate. The age, sex, race, and ethnicity of the study participants are described in Tables S1 and S3. Because of the small number of study participants and the predominance of males study persons in the reported clinical trial, we were unable to assess the influence of sex and gender on study outcomes. To qualify, prospective participants had to have normal standard hematology and clinical chemistry tests, had to be negative for serologic markers of chronic hepatitis B and C, and had to have no evidence for cardiac ischemia in a standard protocol stress echocardiography. Other entry and exclusion criteria are detailed in the study protocol.

#### Study medication

Sequential cohorts of study participants were enrolled in a dose-escalation study. The first two dosing cohorts (stages 1 and 2) served primarily to assess the safety of panobinostat administration together with PEG-IFN-α2a; the third dose cohort (stage 3) had a larger sample size to allow for comparison of the effects of panobinostat plus PEG-IFN-α2a versus panobinostat or PEG-IFN-α2a alone on HIV-1 reservoir cells. Panobinostat was used as immediate-release solid tablets at a dose of 5 mg panobinostat for stage 1, 10 mg panobinostat for stage 2, and 15 mg panobinostat for stage 3. The drug was manufactured and provided free of charge by Novartis Pharmaceutical Corporation during the initial stage of the study and by Secura Bio during later stages. PEG-IFN-α2a was administered at a fixed dose of 180μg and provided free of charge by Genentech Incorporation. The pharmaceutical companies providing study medications were offered an opportunity to review the manuscript but did not contribute to study design and performance, data analysis or article preparation.

#### Randomization

Participants were randomized by a computerized algorithm to treatment Arm A or treatment Arm B or treatment Arm C. Participants in treatment Arm A (n=2 participants in stages 1 and 2; n=4 participants in stage 3) received treatment with panobinostat only, and participants in treatment Arm B (n=6 participants in stage 1 and 2; n=9 participants in stage 3) received combined treatment with panobinostat and PEG-IFN-α2a. Participants in Arm C (n=4 in stage 3) received treatment with PEG-IFN-α2a only. Randomization occurred on day 0 of the study, immediately prior to administration of the first dose of the study medication. Participants were informed about the randomization decision, and open-label study medication was administered.

#### Study Procedures

After obtaining informed consent, all study participants had a history and physical exam performed and had laboratory tests to confirm they meet all inclusion and none of the exclusion entry criteria. Study medications were administered during one week, followed by three weeks of observation. One oral dose of panobinostat was administered every other day for one week in participants randomized to group A and B, resulting in a total treatment with three doses of panobinostat. One subcutaneous injection with pegylated Interferon-α2a was administered at the start of the week-long treatment course to participants randomized to group B and C. ART was continued during the entire study period in all study participants. Participants underwent close monitoring for side effects during the entire time of study participation. Blood samples were obtained for immunological and virological assays at study baseline and at selected time points during the treatment and observation periods as summarized in the study protocol.

#### Safety data review

All safety events were documented by study staff and submitted for review to an independent (non-investigator) safety monitoring committee, to the local IRB and the FDA. The intensity of adverse events was graded according to the NIH Division of AIDS “System for Grading the Severity of Adult Adverse Events” (Version 2.0, November 2014). After completion of stages 1 and 2, an interim safety review by a non-investigator safety monitoring committee was performed; study progression to the next higher panobinostat dose was supported in each case by the safety monitoring committee.

### Method details

#### Collection, isolation and cryopreservation of cells

Peripheral blood samples were collected in ACD tubes and processed to mononuclear cell isolation by Ficoll density gradient centrifugation. Isolated peripheral blood mononuclear cells were cryopreserved according to standard protocols of the AIDS Clinical Trials Group.

#### Isolation of CD4^+^ T cells

Immunomagnetic enrichment of CD4^+^ T cells was performed using the EasySep Human CD4^+^ T cell isolation Kit (StemCell, #17952) product, or the Dynabeads CD4 positive isolation kit (Invitrogen, #11145D) using the manufacturer’s protocol.

#### Flow cytometry

PBMCs were thawed, stained with LIVE/DEAD Blue Viability Dye (Invitrogen) for 15 minutes and subsequently preincubated for 10 minutes with FcR blocking reagent (Miltenyi). Afterward, cells were incubated for 30 minutes at 4°C with different combinations of appropriately titrated antibodies directed against surface and intracellular markers listed in Table S6. To analyze the acetylated histone H3 expression, following the surface staining, cells were fixed with PBS + 1% PFA (Affymetrix) for 15 minutes at 4°C, permeabilized with 0.1% Triton X-100 (Millipore) in PBS for 10 minutes at room temperature, and incubated with PBS + 10% FBS for 20 minutes at room temperature. The cells were then stained with rabbit anti-acetyl histone H3 (Millipore) in the presence of normal rabbit serum (Millipore) for 1 hour at room temperature, followed by incubation with FITC-conjugated goat Anti-Rabbit antibody (Millipore) for 1 hour at room temperature. To analyze HIV-1–specific T cell responses, PBMCs were rested in R10 medium for 4 hours at 37°C in 5% CO2 and then incubated with an HIV-1 consensus clade B Gag peptide pool (mix of 121 overlapping 15-mer peptides at 2 μg/ml for each peptide, NIH AIDS Reagent Program #12756) in the presence of 1 μg/ml anti-CD28 and anti-CD49d (BD Biosciences) and antibodies against CD107a and CD107b (clones H4A3 and H4B4, respectively, BD Biosciences). DMSO and 0.4 μg/ml SEB (Sigma-Aldrich) were used as negative and positive controls, respectively. After 1 hour of incubation, 5 μg/ml Brefeldin A (BioLegend) and 1 μg/ml Monensin (BD Biosciences) were added, and cells were incubated for an additional 12 hours, followed by flow cytometry staining protocols. Subsequently, the cells were fixed in 2% paraformaldehyde in PBS (Affymetrix) and acquired on a BD LSRFortessa cytometer (BD Biosciences). Unstimulated controls were run for each sample and subtracted as background. Data were analyzed using FlowJo v.10.5.3 software (Tree Star LLC) and using the Simplified Presentation of Incredibly Complex Evaluations (SPICE) software (version 6.0).

#### DNA/RNA extraction

Isolated CD4^+^ T cells were subjected to DNA and RNA extraction using commercial kits (GeneElute RNA/DNA/Protein Purification Plus kit, Sigma Aldrich, #RDP300). DNA extraction was performed using commercial kits (Qiagen DNeasy Blood and Tissue Kit, #69504).

#### Integration Site Analysis

Integration sites were obtained using integration site loop amplification (ISLA), using a protocol previously described.^53^ Genomic DNA extracted from 1-3 million isolated CD4^+^ T cells was used as a template. PCR products of the ISLA reaction were subjected to next-generation sequencing using Illumina MiSeq. MiSeq paired-end FASTQ files were demultiplexed; small reads (142 bp) were then aligned simultaneously to the human reference genome GRCh38 and HIV-1 reference genome HXB2 using bwa-mem.^93^ Biocomputational identification of integration sites was performed according to previously-described procedures.^53^ The final list of integration sites and corresponding chromosomal annotations was obtained using Ensembl (v109, www.ensembl.org), the UCSC Genome Browser (www.genome.ucsc.edu), and GENCODE (v43, www.gencodegenes.org). Repetitive genomic sequences harboring HIV-1 integration sites were identified using RepeatMasker (www.repeatmasker.org). A subset of integration sites were obtained using ligation-mediated PCR (LM-PCR), using a previously described experimental and biocomputational protocol.^54^ A list of all integration sites identified in this study is provided in Table S7. ZNF genes were identified based on the classification by the HUGO Gene Nomenclature Committee (HGNC; www.genenames.org; accessed in February 2023).^96^ A list of KRAB-ZNF genes was obtained from a previously published manuscript.^97^

#### Intact Proviral DNA Assay (IPDA)

The standard IPDA protocol described previously^41^ was used. An average of 7.6x10^5^ CD4^+^ T cells were analyzed per sample, and the mean of multiple replicates normalized per million CD4^+^ T cells was calculated. When viral copies were undetectable, data were reported as “limit of detection” (LOD, empty symbol), calculated as 1 copy per maximum number of cells tested without target identification.

#### Near full-length proviral sequencing

Near full-length proviral sequencing (FLIP-seq) was conducted as described in our prior work.^5^ DNA diluted to single genome levels based on Poisson distribution statistics and IPDA results was subjected to single-genome amplification using Invitrogen Platinum Taq and nested primers spanning near-full-length HIV-1 (HXB2 coordinates 638-9632). Primers were previously published.^5^ PCR products were visualized by agarose gel electrophoresis. Amplification products were individually subjected to Illumina MiSeq sequencing at the MGH DNA Core facility. Resulting short reads were de novo assembled and aligned to HXB2 to identify large deleterious deletions, out-of-frame indels, premature/lethal stop codons, internal inversions, or packaging signal deletions, using an automated in-house pipeline (https://github.com/BWH-Lichterfeld-Lab/Intactness-Pipeline). Presence/absence of APOBEC-3G/3F–associated hypermutations was determined using the Los Alamos HIV Sequence Database Hypermut 2.0 program.^92^ Viral sequences that lacked all mutations listed above were classified as “genome-intact.” Multiple sequence alignments were performed using MUSCLE.^91^ Phylogenetic distances between sequences were examined using Clustal X–generated neighbor-joining algorithms.^98^ When indicated, full-genome amplification was preceded by a multiple displacement amplification step involving phi29 polymerase (QIAGEN REPLI-g Single Cell Kit, catalog 150345), as described for the MIP-Seq assay in our previous work^12^; subsequently, DNA from each well was split and separately subjected to viral sequencing and integration site analysis by ISLA. Sequences were considered clonal if they were completely identical or if they differed by a maximum of 1 bp.

#### Analysis of CD4^+^ T cell-associated HIV-1 RNA

Total RNA extracted from CD4^+^ T cells was first subjected to DNase treatment (DNase I, Invitrogen, #18068015) for 15 min at room temperature, followed by incubation with EDTA for 10 min at 65°C for enzyme inactivation. The RT reaction was performed with SuperScript III RT in a buffer containing 10mM dNTPs, 0.1M DTT, RNAse out (40U/μl) with primers described before.^35^^,^^37^ RT reactions were performed at 50°C for 50 min, followed by an inactivation step at 85°C for 5 min. Then 3.5μl of RNaseH was added and the RT product was incubated for 20 min at 37°C. The RT product was then submitted for droplet digital PCR as described before.^37^ Samples were tested in quadruplicate, droplets were read and analyzed using the QuantaSoft software. The transcripts were normalized to 1μg of RNA.

#### Cut & Run sequencing

A standard CUT&RUN sequencing protocol was used^62^ with minor modifications. Briefly, isolated CD4^+^ T cells were bound to concanavalin A–coated magnetic beads (Bangs Laboratories), followed by cell permeabilization using 0.01% digitonin (Millipore) in wash buffer. Primary antibodies against intracellular H3K27ac and H3K27me3 (Cell Signaling Technology) were added, and cells were incubated at 4°C overnight. Guinea pig anti-rabbit IgG antibody (Antibodies-online) was used as a negative control. The next day, fusion protein CUTANA pA/G-MNase (20×) (Epicypher) was added, and cells were incubated at 4°C for 1 hour, followed by chromatin digestion using 100 mM CaCl 2 (Thermo Fisher Scientific). DNA fragments were extracted using phenol chloroform (Invitrogen) and the extracted DNA was eluted in 1 mM Tris-HCl pH 8.0 (Thermo Fisher Scientific) supplemented with 0.1 mM EDTA (Sigma-Aldrich). DNA libraries were prepared using NEBNext Ultra II DNA Library Prep Kit for Illumina Sequencing (New England Biolabs). The DNA library concentration and quality were measured by the Qubit 1× dsDNA HS kit (Life Technologies) and the D1000 High Sensitivity TapeStation (Agilent), respectively. Next-generation sequencing was performed using NextSeq 500/550 High Output v2.5 kit (75 cycles) (Illumina) on a NextSeq 500 Instrument (Illumina). Adapters and low-quality reads were trimmed using Trimmomatic^99^ and aligned to the human genome (GRCh38) using Bowtie2.^100^ Integration sites in regions blacklisted in the ENCODE Data Analysis Center were excluded from the analysis.^101^ Peak calling was implemented using MACS2.^87^ For visualization, the coverage profile was calculated using DeepTools.^88^ Differential binding analysis was performed using DiffBind.^89^The CUT&RUN sequencing data are available in Gene Expression Omnibus.

#### Gene expression analysis by RNA-seq

Total RNA from isolated CD4^+^ T cells was extracted and purified using the PicoPure RNA Isolation Kit (Applied Biosystems). Subsequently, RNA-seq libraries were generated as previously described.^102^ The whole transcriptome amplification (WTA) and tagmentation-based library preparation were performed using Nextera XT (Illumina), followed by sequencing on a NextSeq 2000 Instrument (Illumina). Sequences from RNA-seq were aligned to the human genome (GRCh38) using STAR^103^ and quantified using RSEM.^94^ Raw counts at gene or isoform levels were normalized using External RNA Controls Consortium spiked-in controls through RUV-seq,^104^ and then used for differential gene expression analysis with DESeq2.^90^ Transcripts per million (TPM) values were used for downstream analysis. Differentially expressed genes (DEGs) from the RNA-seq data set were analyzed using the IPA (Qiagen)^105^ and the Database for Annotation, Visualization, and Integrated Discovery (DAVID) v6.8.^95^

#### 3D chromosomal contact data

3D chromosomal contact data from CD4^+^ T cells generated using the Hi-C, previously collected from 3 participants under ART,^13^ were used. For data analysis, reads from central memory, effector memory, and total CD4^+^ T cells were combined to generate a pooled dataset with an overrepresentation of reads from memory cells to account for the fact that most HIV-1-infected cells are included in the memory cell compartment.^106^ Significant intra-chromosomal interactions were called using FitHiC2^107^ with Knight-Ruiz matrix balancing^108^ and FDR-adjusted p-values < 0.05 as a cutoff^107^^,^^109^ at a binning resolution of 20kb.

### Quantification and statistical analysis

Data are presented as histograms, single data scatter plots, circos plots, bar diagrams, line graphs, and box and whisker plots, showing the median, the 25% and 75% percentiles, and maximum/minimum values. Differences were tested for statistical significance using Mann-Whitney U tests (two-tailed), Chi-Square tests, Wilcoxon matched-pairs signed rank tests (two-tailed), Friedman tests, or Kruskal-Wallis tests, as appropriate. Adjustments for multiple comparison testing were performed using Dunn’s multiple comparison tests or using false discovery rate calculations.^110^ Correlations were determined using Spearman correlation coefficient. p-values of <0.05 were considered significant. Analysis was performed using Prism (GraphPad Software, Inc.), Python (Python Software Foundation), and R (R Foundation for Statistical Computing).^111^

### Additional resources

The study was approved by the local IRB (protocol number 2015P000858) and was conducted under US FDA IND 123446 and NIAID DAIDS-ES ID 12049; the study was registered at https://clinicaltrials.gov under NCT02471430.

## Supplemental information

Document S1. Tables S1–S6

Table S7. List of proviral integration sites identified in this study, related to Figure 5
