# Supplementary material for: Selection of epigenetically privileged HIV-1 proviruses during treatment with panobinostat and interferon-α2a
Source: Cell. 2024 Feb 29;187(5):1238–1254.e14. doi: 10.1016/j.cell.2024.01.037 (PMC10903630; doi:10.1016/j.cell.2024.01.037)
Supplement: Document S1. Tables S1–S6 [file mmc1.pdf]

Cell, Volume *187*

Supplemental information

Selection of epigenetically privileged HIV-1 proviruses during treatment with panobinostat and interferon-α2a

Marie Armani-Tourret, Ce Gao, Ciputra Adijaya Hartana, WeiWei Sun, Leah Carrere, Liliana Vela, Alexander Hochroth, Maxime Bellefroid, Amy Sbrolla, Katrina Shea, Theresa Flynn, Isabelle Roseto, Yelizaveta Rassadkina, Carole Lee, Francoise Giguel, Rajeev Malhotra, Frederic D. Bushman, Rajesh T. Gandhi, Xu G. Yu, Daniel R. Kuritzkes, and Mathias Lichterfeld

		Panobinostat (n=4)	Panobinostat + IFNα2a (n=9)	IFNα2a (n=4)
Age, years (min-max)		49.5 (41-60)	40.4 (26-58)	43.25 (35-47)
Male, n (%)		3 (75)	9 (100)	2 (50)
Ethnicity, not Hispanic or Latino, n (%)		2 (50)	4 (44.4)	3 (75)
Race	White, n (%)	3 (75)	8 (88.8)	2 (50)
	Asian, n (%)	0 (0)	1 (11.2)	0 (0)
	Black or African American, n (%)	1 (25)	0 (0)	2 (50)
Risk group	MSM, n (%)	3 (75)	9 (100)	2 (50)
	Heterosexual Transmission, n (%)	1 (25)	0 (0)	1 (25)
	Unknown, n (%)	0 (0)	0 (0)	1 (25)

Supplemental Table 1 (related to Figure 1): Clinical and demographical characteristics of the study participants in Stage 3.

**Phase III**	Panobinostat (n=4)	Panobinostat + IFNα2a (n=9)	IFNα2a (n=4)
Any adverse event, n %	3 (75%)	9 (100%)	4 (100%)
Any adverse event grade 3 or higher, n %	0 (0%)	1 (11.1%)	0 (0%)
Body aches/achiness		4 (gr. 2) (44.4%)	1 (gr. 1) (25%)
Fatigue		6 (3 gr. 2; 3 gr. 1) (66.6%)	1 (gr. 1) (25%)
Paresthesia		1 (gr. 1) (11.1%)	
Nausea	1 (gr. 1) (25%)	2 (gr. 1) (22.2%)	1 (gr.1) (25%)
Fever		2 (gr. 2) (22.2%)	
Chills		1 (gr. 2) (11.1%)	
Diarrhea/Loose stool	2 (gr. 1) (50%)	4 (gr. 1) (44.4%)	
Leukopenia		3 (1 gr. 3; 2 gr. 1) (33.3%)	
Vomiting		1 (gr. 2) (11.1%)	
Increased serum creatinine	1 (gr. 1) (25%)		
ECG changes	1 (NA) (25%)		
Headache		1 (gr. 1) (11.1%)	1 (gr. 2) (25%)
Rash		1 (gr. 2) (11.1%)	

Supplemental Table 2 (related to Figure 1): Adverse events in Stage 3

		Panobinostat (n=4)	Panobinostat + IFNα2a (n=12)
Age, years (min-max)		55.8 (47.5-62.2)	51.3 (37.7-64.1)
Male, n (%)		4 (100)	8 (66.6)
Ethnicity, not Hispanic or Latino, n (%)		2 (50)	9 (75)
Race	White, n (%)	3 (75)	7 (58.3)
	Asian, n (%)	0 (0)	1 (8.3)
	Black or African American, n (%)	1 (25)	4 (33.3)
Risk group	MSM, n (%)	4 (100)	8 (66.6)
	Heterosexual Transmission, n (%)	0 (0)	4 (33.3)
	Unknown, n (%)	0 (0)	0 (0)

Supplemental Table 3 (related to Figure 1): Clinical and demographical characteristics of the study participants in Stages 1 and 2.

**Phase I**	Panobinostat (n=2)	Panobinostat + IFNα2a (n=6)
Any adverse event, n %	1 (50%)	5 (83.3%)
Any adverse event grade 3 or higher, n %	0 (0%)	0 (0%)
Body aches		4 (3 gr.1; 1 gr.2) (66.6%)
Fatigue		2 (1 gr.1; 1 gr.2) (33.3%)
Dizziness		1 (1 gr.1) (16.6%)
Nausea		1 (1 gr.1) (16.6%)
Fever		2 (1 gr.1; 1 gr.2) (33.3%)
Lightheadedness		1 (1 gr.1) (16.6%)
Leukopenia		2 (2 gr.2) (33.3%)
Stomach cramping		1 (1 gr.1) (16.6%)
Diarrhea/Loose stool		1 (1 gr.1) (16.6%)
Abdominal Bloating	1 (1 gr.1) (50%)	1 (1 gr.1) (16.6%)
Headache		2 (2 gr.) (33.3%)

Supplemental Table 4 (related to Figure 1): Adverse events in Stage 1

**Phase II**	Panobinostat (n=2)	Panobinostat + IFNα2a (n=6)
Any adverse event, n %	0 (0%)	6 (100%)
Any adverse event grade 3 or higher, n %	0 (0%)	2 (33.3%)
Body aches		2 (1 gr.1; 1 gr.2) (33.3%)
Fatigue		1 (1 gr.2) (16.6%)
Fever		1 (1 gr.1) (16.6%)
Chills		4 (3 gr.1; 1 gr.2) (66.6%)
Lightheadedness		3 (2 gr.1; 1 gr.2) (50%)
Low ANC		2 (2 gr.3) (33.3%)
Diarrhea/Loose stool		1 (1 gr.1) (16.6%)
Abdominal Bloating		2 (1 gr.1; 1 gr.2) (33.3%)
Headache		1 (1 gr.1) (16.6%)
Leukopenia		1 (1 gr.2) (16.6%)
Superficial punctate keratitis		1 (1 gr.2) (16.6%)
Hypoglycemia non fasting		1 (1 gr.1) (16.6%)
Elevated ALT/AST		1 (1 gr.1) (16.6%)
Lower back pain		1 (1 gr.2) (16.6%)
Lower leg muscles aches		1 (1 gr.1) (16.6%)
Dry cough		1 (1 gr.2) (16.6%)
General body weakness		1 (1 gr.2) (16.6%)
Mild sweats		1 (1 gr.1) (16.6%)

Supplemental Table 5 (related to Figure 1): Adverse events in Stage 2

Acetylated H3 Panel					
		Fluorochrome	Clone	Reference	Company
1	CD95	PE/Dazzle594	DX2	305634	BioLegend
2	CD3	AF700	UCHT1	300424	BioLegend
3	CCR7	AF647	G043H7	353218	BioLegend
4	CD45RA	BV711	HI100	304138	BioLegend
5	CD8a	BV510	RPA-T8	301048	BioLegend
6	CD4	BUV395	RPA-T4	564724	BD Biosciences
**NK cell Panel**					
1	NKp30	PerCP-Cy5.5	P30-15	325216	BioLegend
2	NKG2A (CD159a)	Viobright-FITC	REA110	130-105-646	Miltenyi
3	NKp46	PE-Cy7	9.00E+02	331916	BioLegend
4	NKG2D (CD159d)	PE/Dazzle594	1D11	320828	BioLegend
5	Siglec7 (CD328)	PE	REA214	130-100-983	Miltenyi
6	CD3	APC-Cy7	HIT3	300318	BioLegend
7	CD14	APC-Cy7	HCD14	325620	BioLegend
8	CD19	APC-Cy7	B4	302218	BioLegend
9	CD38	AF700	HIT2	303524	BioLegend
10	NKG2C (CD159c)	APC	134591	FAB138A-100	R&D Systems
11	PD1	BV785	EH12.2H7	135225	BioLegend
12	CD56	BV711	HCD56	318336	BioLegend
13	CD161	BV650	DX12	563864	BD Biosciences
14	CD57	PB	HCD57	322316	BioLegend
15	CD16	BUV395	3G8	563785	BD Biosciences
**DC/Monocyte Panel**					
1	CD3	PerCP-Cy5.5	HIT3a	300328	BioLegend
2	CD19	PerCP-Cy5.5	HIB19	302230	BioLegend
3	CD20	PerCP-Cy5.5	2H7	302326	BioLegend

4	CD56	PerCP-Cy5.5	HCD56	318322	BioLegend
5	CD40	FITC	5C3	334306	BioLegend
6	CD1c (BDCA-1)	PE-Cy7	L161	331516	BioLegend
7	ICOSL (CD275)	PE/CF594	2D3/B7-H2	564277	BD Biosciences
8	HLA-DR	PE	L203	FAB4869P-100	R&D Systems
9	CD83	APC-Cy7	HB15e	305330	BioLegend
10	CD14	AF700	63D3	367114	BioLegend
11	CD141 (BDCA-3)	APC	1A4	564123	BD Biosciences
12	CCR7	BV785	G043H7	353230	BioLegend
13	PD-L1 (CD274)	BV711	29E.2A3	329722	BioLegend
14	CD86	BV650	IT2.2	305428	BioLegend
15	CD123	BV510	6H6	306022	BioLegend
16	CD11c	BV421	Bu15	337226	BioLegend
17	CD16	BUV395	3G8	563785	BD Biosciences
**HIV-1 specific T cell Panel**					
1	PD1 (CD279)	PerCP-Cy5.5	EH12.2H7	329914	BioLegend
2	CD3	PE-Cy7	UCHT1	300420	BioLegend
3	CD8a	APC-Cy7	RTA-T8	301016	BioLegend
4	CD4	BUV395	RPA-T4	564724	BD Biosciences
5	Perforin	FITC	B-D48	353310	BioLegend
6	Eomes	PE-Efluor610	WD1928	61-4877-42	eBioscience
7	T-bet	PE	4B10	644810	BioLegend
8	Granzyme A	AF700	CB9	507210	BioLegend
9	IFNϒ	AF647	4S.B3	502516	BioLegend
10	IL-2	BV650	MQ1-17H12	500334	BioLegend
11	Granzyme B	BV510	GB11	563388	BD Biosciences
12	TNFα	BV421	MAb11	502932	BioLegend

Supplemental Table 6 (related to STAR Methods): List of antibodies used in flow cytometry experiments
